# Systematic Identification and Functional Validation of New snoRNAs in Human Muscle Progenitors

**DOI:** 10.3390/ncrna7030056

**Published:** 2021-09-13

**Authors:** Baptiste Bogard, Claire Francastel, Florent Hubé

**Affiliations:** UMR7216 Épigénétique et Destin Cellulaire, CNRS, Université de Paris, F-75013 Paris, France; baptiste.bogard@univ-paris-diderot.fr

**Keywords:** intron, snoRNA, medium RNA-seq, gene annotation, nonsense mediated decay, nucleolar, snoRNA host-gene, intergenic snoRNA, human muscle progenitors

## Abstract

Small non-coding RNAs (sncRNAs) represent an important class of regulatory RNAs involved in the regulation of transcription, RNA splicing or translation. Among these sncRNAs, small nucleolar RNAs (snoRNAs) mostly originate from intron splicing in humans and are central to posttranscriptional regulation of gene expression. However, the characterization of the complete repertoire of sncRNAs in a given cellular context and the functional annotation of the human transcriptome are far from complete. Here, we report the large-scale identification of sncRNAs in the size range of 50 to 200 nucleotides without a priori on their biogenesis, structure and genomic origin in the context of normal human muscle cells. We provided a complete set of experimental validation of novel candidate snoRNAs by evaluating the prerequisites for their biogenesis and functionality, leading to their validation as genuine snoRNAs. Interestingly, we also found intergenic snoRNAs, which we showed are in fact integrated into candidate introns of unannotated transcripts or degraded by the Nonsense Mediated Decay pathway. Hence, intergenic snoRNAs represent a new type of landmark for the identification of new transcripts that have gone undetected because of low abundance or degradation after the release of the snoRNA.

## 1. Introduction

The continuous progress in the annotation of mammalian genomes first led to the striking conclusion that they are mostly non-coding, with only 2% having information to encode proteins [1]. In addition, sequencing of their transcriptional outputs revealed that almost 90% of these genomes is transcribed, leading to the remarkable conclusion that most of the RNA content of a mammalian cell consists of so-called non-coding RNAs (ncRNAs) [2]. Classically, ncRNAs are classified as long or short ncRNAs (lncRNAs or sncRNAs) depending on their length and a cutoff of 200 nt [3]. It is now admitted that they add layers to regulatory circuitries through their implication in most cellular processes, ranging from chromatin remodeling, transcription, splicing and translation to shaping nuclear architecture [4]. 

We have also reported that a single transcription unit could in fact generate multiple RNA species, including long or short coding, or non-coding RNAs depending on alternative splicing (AS) events of introns, whereby introns can be either spliced or retained in the host transcript [5,6,7,8]. Hence, the AS of introns is a versatile “developmental switch” that provides a certain plasticity to eukaryotic genomes, allowing us to diversify their transcriptional output depending on the cellular context or environmental cues and, ultimately, to control fate choices of progenitor cells. Although AS of introns remains poorly documented in mammals, it is far from anecdotal since introns account for almost half of the human genome and could represent a large repertoire of candidates it has already predicted [9,10,11]. An important consequence is that splicing defects that characterize many human diseases such as cancers or the Myotonic Dystrophy type 1 (DM1), caused by a toxic RNA-based sequestration of the splicing factor Muscleblind Like Splicing Regulator 1 (MBNL1), may have a much broader impact than just affecting the production of proteins [12].

Being removed from the pre-mRNA to allow for the formation of mature mRNA, the intron lariat is then disconnected by the Debranching enzyme 1 (DBR1) and rapidly degraded within minutes after its excision. Intrinsically non-coding and with a short half-life, a subset of introns nevertheless shows high sequence conservation across closely related species, suggesting functional constraints on intronic sequences throughout evolution [13]. Many studies have now shown that introns can shelter sncRNAs in which biogenesis is strictly dependent on both transcription and splicing of their host gene, with the most studied examples being the small nucleolar RNAs (snoRNAs) [14,15]. During splicing, snoRNA-hosting introns are protected from degradation by exonucleases through the recruitment of specific protein factors, which allows for further snoRNA maturation [16].

We previously referred to regulatory ncRNAs originating from intron splicing as Short Intron-Derived small ncRNAs (SIDs) [5]. SIDs include some non-canonical miRNAs such as mirtrons, simtrons and agotrons, and all snoRNAs, which represent the largest group of SIDs in humans [17,18,19,20]. These ncRNAs, 50–300 nt in length, mainly operate as guides to mediate posttranscriptional modifications on other ncRNAs such as ribosomal RNAs (rRNAs) and small nuclear RNAs (snRNAs) [21,22]. SnoRNAs are classified into two classes based on their secondary structure and specific sequence motifs, named C/D box snoRNAs and H/ACA box snoRNAs. C/D box snoRNAs possess two sequence motifs, the C box (RUGAUGA, R = A or G) and the D box (CUGA). They are part of a larger RiboNucleoProtein (RNP) complex called snoRNP containing Nucleolar proteins (NOP) NOP56 and NOP58, 15.5 kDa protein and Fibrillarin (FBL), in which the snoRNA molecule recognizes its target in a base-pairing manner and the methyltransferase FBL catalyzes the 2′O-methylation [23,24]. H/ACA box snoRNAs are characterized by the presence of an H box (ANANNA, N = A, C, G or U) and an ACA box. They are in complex with the proteins Glycine Arginine Rich protein 1 (GAR1), Non-Histone chromosome Protein 2 (NHP2), NOP10 and Dyskerin (DKC1). H/ACA box snoRNPs are responsible for pseudouridylation through the pseudouridine synthase DKC1 [23,25]. As suggested by their name, snoRNAs accumulate and operate in nucleoli [26,27]. A subset of snoRNAs, named small Cajal Body-specific RNAs (scaRNAs), possess a H/ACA, a C/D box or both and can guide both 2′O-methylation and pseudouridylation of snRNAs in Cajal Bodies (CBs) [28,29]. Indeed, in contrast with other snoRNAs, scaRNAs have a particularity in that they possess a Cajal body-specific localization signal, the CAB box, and co-localize with Coilin (COIL) in CBs [28,30,31]. 

Numerous studies have expanded the repertoire of the human transcriptome with new small ncRNAs [27,32,33,34,35]. Thus far, biocomputational predictions from small RNA-sequencing (RNA-seq) analysis have identified either new miRNAs or new snoRNAs [27,32,33,34,35]. We have also identified novel SIDs, namely non-canonical miRNAs, through biocomputational predictions using a palindromic sequence search-based approach dedicated to the identification of intronic pre-miRNAs [5]. For the identification of snoRNAs, specific sequencing methods were designed based on the use of thermostable group II intron reverse transcriptase sequencing (TGIRT-seq) that allows for the detection of highly structured RNAs such as H/ACA box snoRNAs and tRNAs [36] or by photoreactive nucleotide-enhanced crosslinking and immunoprecipitation (PAR-CLIP) of the core proteins of the snoRNPs followed by sequencing [35]. 

In order to capture the full repertoire of small ncRNAs without a priori knowledge of their biogenesis, structure and genomic origin, we designed a “medium” RNA-seq on total RNAs with a size range of 50–200 nt, compatible with the known size of sncRNAs precursors, and depleted in rRNAs and poly(A^+^) RNAs to enrich sncRNAs independently of their host genes while eliminating abundant mature miRNAs. We applied this strategy to the context of human muscle progenitor cells and uncovered around 400 yet unannotated sncRNAs. Biocomputational predictions revealed that one-third corresponded to snoRNAs, 14% corresponded to new pre-miRNAs and 8% corresponded to snRNAs. Since snoRNAs were assumed to be all annotated, although recent studies have pointed out that it was not quite the case [27,35,36,37,38], we focused on newly identified snoRNA candidates to experimentally validate them by checking their incorporation into snoRNPs and their accumulation in nucleoli. We validated 30 of them as genuine snoRNAs, of which four were surprisingly not located in a gene body, indicative of new unannotated transcription units since snoRNAs mostly originate from intron splicing. 

Altogether, this work provides the identification of a full scope of sncRNAs in a size range that excludes well-characterized and abundant mature miRNAs and piRNAs as well as 5S RNA in a given cell type, followed by a thorough procedure of experimental validation of the candidate new snoRNAs. This study also points out the potential of intergenic snoRNAs as indicators of the presence of not yet annotated transcription units. 

## 2. Results

### 2.1. Discovery of New Unannotated Small Non-Coding RNAs in Human Muscle Cells

**Figure 1:**
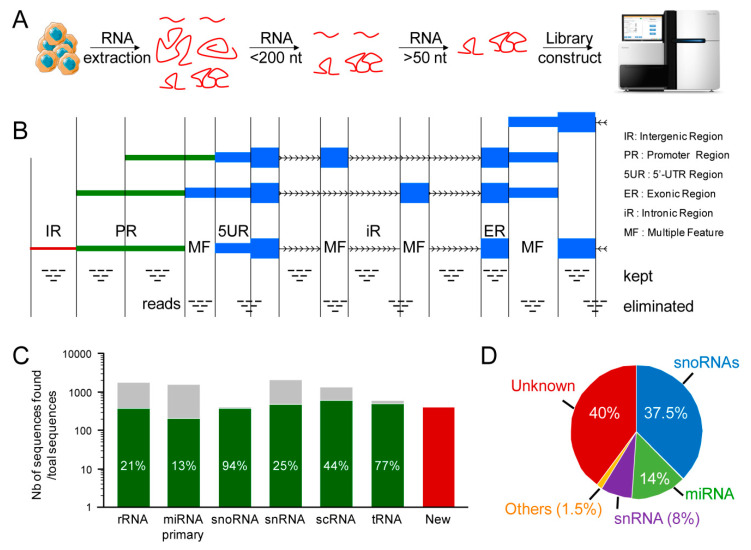
Discovery of new unannotated small non-coding RNAs. (**A**) Schematic representation of the method (see Section 4 for more details). (**B**) Reads that mapped to multiple features, e.g., 5′-UTR and promoter regions, or overlapped with two features for instance, were eliminated from the analysis. Only reads that aligned to unique features were kept for further analysis. (**C**) The number of sequences retrieved (green) out of the total number of known sequences (grey) and the number of new unannotated and yet unknown candidates (red). Only 20% of rRNA was retrieved (there is a rRNA depletion during the preparation of the samples), while 15% of miRNA (the correct proportion of miRNAs for a given cell type) and almost all snoRNA and tRNA were recovered (almost all were ubiquitously expressed). In addition, 400 new candidates were retrieved. (**D**) The percentage of candidates showing homology with already known sequences, sorted by families. For instance, 37.5% of newly identified small ncRNAs showed sequence homology to known snoRNAs. IR, Intergenic Region (red); PR, Promoter Region (green); 5UR, 5′-UTR Region (thin blue); ER, Exonic Region (thick blue); iR, intronic Region (black); MF, Multiple Feature (empty); rRNA, ribosomal RNA; miRNA, microRNA; snoRNA, small nucleolar RNA; snRNA, small nuclear; scRNA, small cytoplasmic RNA; tRNA, transfer RNA; New, new unannotated and yet unknown small RNA candidates.

To systematically identify small ncRNA candidates without a priori on their genomic location, secondary structure and biogenesis and independently of their host transcript, we isolated ribo- and poly(A)-depleted RNAs extracted from human myoblasts (MBs) and their differentiated myotube (MT) counterparts. The RNAs were size-fractionated to isolate the fraction > 50 and <200 nt in length and further sequenced using the medium RNA-seq procedure described in the Material and Methods section and in Figure 1A. We filtered out and separately analyzed all of the reads mapping to multiple gene features (i.e., exons, introns, promoters, intergenic, 5′UTR and/or 3′UTR, extracted from UCSC Table browser) (Figure 1B) to already annotated small ncRNAs or to transcripts from repeated sequences (rmsk from UCSC Table browser). To validate this approach, we verified that all of the annotated snoRNAs and tRNAs as well as the correct proportion of miRNAs for a given cell type (around 15%) [39] were retrieved from the medium RNA-seq (Figure 1C). We then focused on clusters of at least 20 reads mapping to intergenic or intronic regions, which therefore could represent new small ncRNA candidates. We uncovered almost 400 new unannotated small ncRNAs for which the mean size was 143 nt (median 126 nt), with a minimum of 52 nt and a maximum of 772 nt, with the few sncRNAs > 200 nt in size (13 were > 400 nt) probably originating from a leakage of the size fractionation procedures. Interestingly, 262 of the new candidates were intronic, whereas 137 were intergenic (Supplementary Table S1). Blast analysis of these 399 sncRNA candidates retrieved 14 homologs of known miRNAs, 26 of snRNAs, 114 of snoRNAs, and 1 of tRNA (Supplementary Tables S1 and S2). However, 239 new candidates were not related to any previously annotated sncRNAs (Figure 1D). We then used dedicated software to predict secondary structures typical of miRNAs or snoRNAs (see Section 4) and found that approximately two thirds of these 239 unannotated candidates potentially represented new snoRNAs, that about 20% corresponded to new miRNAs and that 15% corresponded to new snRNAs (Supplementary Table S1). A large fraction of the new sncRNA candidates (40%, of which 80% are intronic and 20% are intergenic) did not exhibit any of these typical secondary structures or sequence similarity with known sncRNAs, suggesting that they may represent new types of sncRNAs. 

For the rest of the study, we decided to focus on the 175 predicted new snoRNAs. As shown in Supplementary Figure S1, the expression levels of the newly identified snoRNAs were below the expression levels of known snoRNAs by one log, which could explain why they remained undetected in transcriptome studies. Alternatively, classical RNA-seq on RNA > 200 nt or small RNA-seq on RNA < 50 nt are not suitable for the identification of ncRNAs between 50 and 200 nt and snoRNAs could have been missed from such analysis.

### 2.2. The Vast Majority of Newly Identified snoRNAs Are Genuine snoRNAs 

In order to classify the newly identified snoRNA candidates as genuine snoRNAs, they have to satisfy several prerequisites: (i) transcription and splicing of their host gene, (ii) incorporation into snoRNP complexes and (iii) accumulation in nucleoli.

#### 2.2.1. snoRNA Candidates and Their Corresponding Host Genes Are Co-Expressed without Significant Correlation of Their Respective Levels

Since most canonical human snoRNAs are processed from the splicing of introns [40], snoRNA host genes must be expressed in the context of interest. We therefore performed a total RNA-seq (see the Material and Methods section) to validate the transcription of host genes for all snoRNA candidates in human muscle cells (Supplementary Tables S1 and S3). We established that the expression of a given snoRNA candidate was always associated with the expression of its corresponding host gene. Since the number of snoRNA candidates slightly increased during normal muscle differentiation (MB vs. MT) (Supplementary Figure S2), we assessed whether these variations were due to differences in the expression of the corresponding host genes. We intersected data from the medium and total RNA-seq and found that expression levels of less than one third of snoRNA candidates and that of their host genes were correlated, e.g., for the pair #54/LARP4 and the pair #166/SMARCC1 for which the Pearson correlation coefficients (r) were 0.77 and 0.94, respectively (Supplementary Figure S3). In contrast, the expression patterns of the pair #122/*TNPO2* showed no correlation (*r* = 0.04) and the pair #186/*DDX60L* even showed anti-correlated expression levels (*r* = −0.99). As a whole, the mean correlation coefficient of expression levels of the 68 snoRNA candidates and their corresponding host genes was 0.31. These data indicate that the expression levels of the snoRNA candidates do not reflect that of their host genes, suggesting the existence of posttranscriptional mechanisms.

#### 2.2.2. Most of the snoRNA Candidates Associate with Core Proteins of snoRNP Complexes

We randomly selected 21 unannotated intronic sncRNAs predicted as snoRNA candidates to experimentally validate them as genuine snoRNAs (Supplementary Table S1). Among the 21 snoRNA candidates, 9 were predicted to belong to the class of H/ACA snoRNAs, 6 were predicted to belong to the class of C/D snoRNAs and 6 were predicted to belong to the class of scaRNAs. Of note, of the 21 snoRNA candidates, 12 were homologs of already known snoRNAs and 9 did not share homology with any known snoRNA (Supplementary Tables S1 and S2) but were all predicted as snoRNAs by snoReport and/or by snoGPS [38,41].

**Figure 2:**
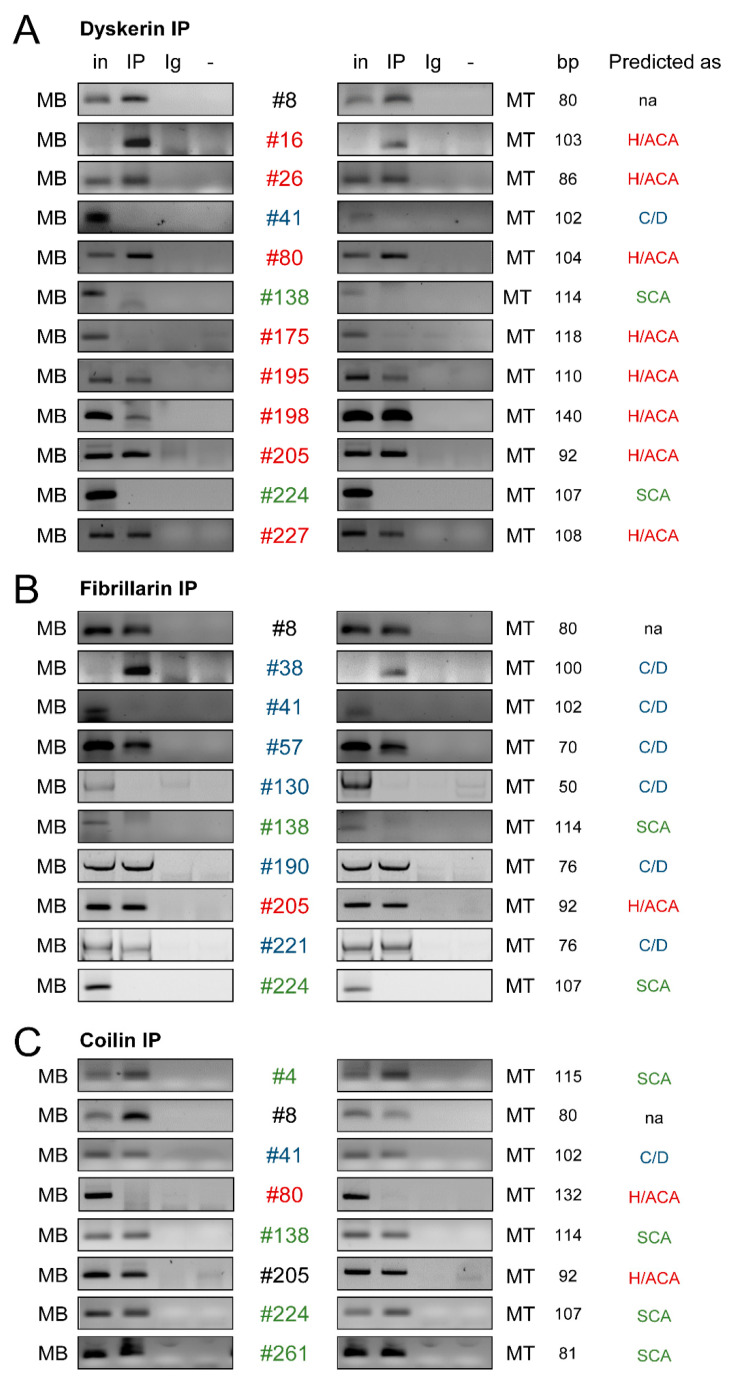
Association of snoRNA candidates with sno-ribonucleoprotein (snoRNP) complexes. IP-PCR against Dyskerin (**A**), Fibrillarin (**B**) and Coilin (**C**) was performed as described in the Material and Methods section to analyze the affiliation of the new snoRNA candidates (#) to their respective snoRNP complexes (n = 2). The class of snoRNA (H/ACA, C/D or SCA) predicted by the webservers is indicated on the right. MB, myoblast; MT, myotube; in, 5% input; IP, immunoprecipitation; Ig, V5 epitope antibody (irrelevant antibody); -, mock PCR; bp, base pair.

To test whether the snoRNA candidates were incorporated into snoRNP complexes, we performed immunoprecipitation (IP) of the Dyskerin, Fibrillarin and Coilin proteins from nuclear extracts of muscle cells, followed by RT-PCR to detect the snoRNA candidates (Supplementary Figure S4A). Western blots controlling the efficiency of immunoprecipitation assays are shown in Supplementary Figure S4B. As a negative control of the experiment, we checked that U6 snRNA was not associated with core proteins of snoRNP complexes, whereas positive controls assessed the association of known snoRNA36B (ACA36B), snoRND88B (HBII-180B) and scaRNA9 (sca9) with their core proteins Dyskerin, Fibrillarin and Coilin (Supplementary Figure S4C–E), respectively. We found that 19 out of the 21 snoRNA candidates tested were in complex with at least one of the core snoRNP proteins (Figure 2). More specifically, of the 9 snoRNA candidates predicted as H/ACA snoRNAs, 8 were found in complex with Dyskerin (Figure 2A). Among the 6 C/D snoRNA candidates (Figure 2B), 5 were found in association with Fibrillarin. Then, all of the candidates predicted as scaRNAs were immunoprecipitated with the Coilin protein (Figure 2C). We also verified that scaRNAs interacting with Coilin did not interact with Dyskerin or Fibrillarin and vice versa. For example, candidate #80, predicted as a H/ACA snoRNA, indeed interacted with Dyskerin but not with Coilin (Figure 2). Interestingly, candidates #138 and #224 predicted as H/ACA scaRNAs were associated with Coilin but not with Dyskerin or Fibrillarin. It was also the case for the candidate #41, which was predicted as a tandem H/ACA-C/D scaRNA and was indeed associated with Coilin but not with Dyskerin or Fibrillarin (Figure 2). In contrast, the candidate #8, which was not predicted as a snoRNA with canonical boxes, was found in complex with Coilin, Dyskerin and Fibrillarin as was already shown for some scaRNAs [30]. Since candidate #8 is located close to SNORA73B in the same intron, this candidate may also resemble a sno-lncRNA where a lncRNA is flanked by two snoRNAs [42]. Hence, we cannot exclude that we co-precipitated SNORA73B, although this supposes that such molecules passed through size fractionation and does not explain the co-precipitation with all three core snoRNPs proteins.

In sum, almost all of the snoRNA candidates that we tested were found associated with snoRNP or scaRNP complexes and could therefore represent new snoRNAs with genuine functions of snoRNAs.

#### 2.2.3. The Majority of snoRNA Candidates Accumulate in Nucleoli

**Figure 3:**
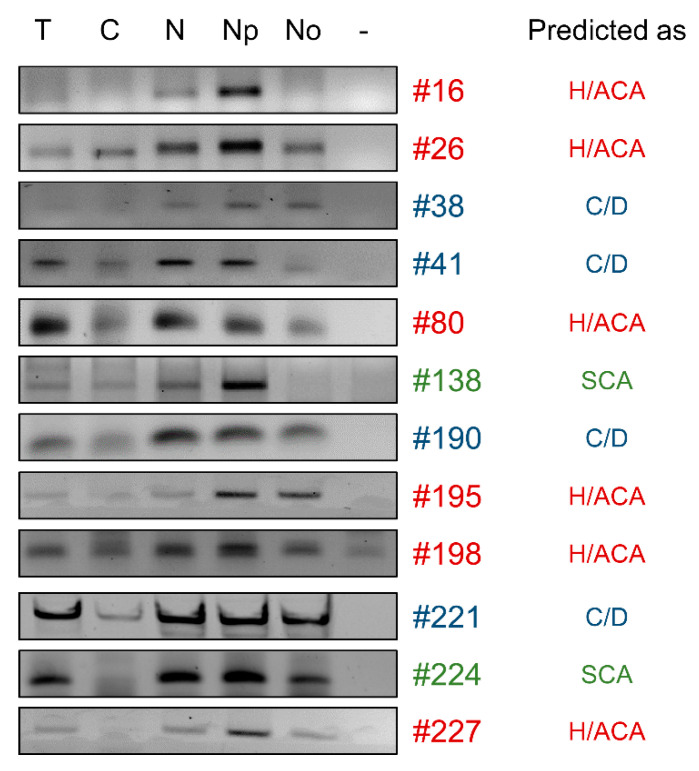
Accumulation of snoRNA candidates in nucleoli. Isolation of the nucleoli by sucrose cushion centrifugation was performed as described in the Material and Methods section. Then, total RNA from each fraction was extracted and localization of snoRNA candidates (#) was assessed by RT-PCR. The class of snoRNA (H/ACA, C/D or SCA) predicted by the webservers is indicated on the right. T, total fraction; C, cytoplasmic fraction; N, nuclear fraction; Np, nucleoplasmic fraction; No, nucleolar fraction; -, mock PCR.

Since canonical snoRNAs are located in nucleoli, in part to guide the modification of rRNAs [23], we assessed the localization of the 12 snoRNA candidates that we found associated with core proteins of snoRNP complexes (Figure 2). We isolated nucleoli from myoblasts by sucrose cushion centrifugation (Supplementary Figure S5A–C) and then detected the snoRNA candidates by RT-PCR [43] (Figure 3 and procedure described in Section 4). Western blots controlling the purity of the nucleoli isolation are shown in Supplementary Figure S5A. As controls of the nuclear fragmentation, we also assessed the localization of known ncRNAs by RT-PCR. As shown in Supplementary Figure S5B, nuclear ncRNAs Nuclear Enriched Abundant Transcript 1 (NEAT1), Metastasis Associated Lung Adenocarcinoma Transcript 1 (MALAT1) and precursor of miRNA-21 (pre-miR-21) were absent from the cytoplasmic and nucleolar fractions, as expected, but enriched in nuclei and nucleoplasm. Known snoRNAs such as SNORD16, SNORD36B and SNORD115-9 were absent from cytoplasmic fractions but enriched in nuclei, nucleoplasm and nucleolar fractions. Finally, as expected, the 18S rRNA was detected in all of the cellular fractions from cytoplasm to nucleoli. Then, out of the 12 snoRNA candidates tested, 9 were enriched in the nucleolar fraction (Figure 3). As for the three snoRNA candidates absent from nucleoli, one was predicted as a scaRNA (#138) and served as a negative control since scaRNAs normally reside in Cajal Bodies [30]. Candidate #41 was predicted as a SNORD although it interacted only with coilin (Figure 2) and may thus be considered a C/D-SCARNA. Candidate #16, predicated as an H/ACA snoRNA, associated with Dyskerin (Figure 2) but was undetectable in the nucleolar fraction (Figure 3). Surprisingly, candidates #26 and #80 were also found in cytoplasmic fractions. In addition, candidates #8, #205 and #224 associated with Coilin but were detected in nucleoli (Figure 2 and Figure 3), suggesting that these candidates could accumulate both in Cajal Bodies and nucleoli as it was recently shown for some scaRNAs, i.e., SCARNA28 [44,45].

Altogether, these results established that most of the snoRNA candidates tested were associated with snoRNPs and accumulated in nucleoli, suggesting that they can be considered as new genuine snoRNAs.

#### 2.2.4. Intergenic snoRNAs as a Hallmark of Yet Unannotated Transcriptional Units

**Figure 4:**
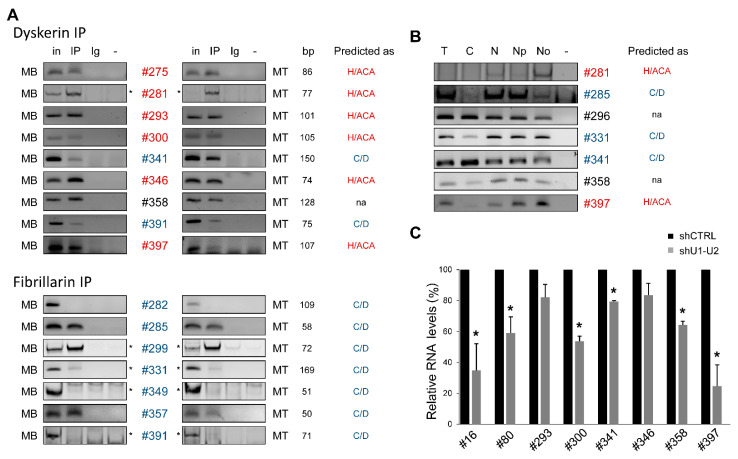
New genuine intergenic snoRNAs are splicing-dependent. (**A**) IP-PCR against Dyskerin and Fibrillarin was performed to analyze the affiliation of the new intergenic snoRNA candidates (#) to their respective snoRNP complex (n = 2). The class of snoRNA (H/ACA, C/D or SCA) predicted by the webservers is indicated on the right. MB, myoblast; MT, myotube; in, 5% input; IP, immunoprecipitation; Ig, V5 epitope antibody (irrelevant antibody); -, mock PCR; bp, base pair. (**B**) Isolation of nucleoli by sucrose cushion centrifugation was performed on MBs to check the accumulation of intergenic snoRNA candidates (#) in nucleoli (for more information, see Figure 3). The class of snoRNA (H/ACA, C/D or SCA) predicted by the webservers is indicated on the right. T, total fraction; C, cytoplasmic fraction; N, nuclear fraction; Np, nucleoplasmic fraction; No, nucleolar fraction; -, mock PCR. (**C**) Total RNA was extracted from MBs transfected with shRNAs targeting snRNP70, PRP8 and U2AF65 (shU1-U2) or Luciferase (shCTRL). Then, new intergenic snoRNA candidates (#) were detected by RT-PCR (n = 3). Intronic snoRNA candidates #16 and #80 known to be splicing-dependent were used as a positive control. Lys-tRNA was used as an invariant control. -, mock PCR. A quantification of the remaining RNA levels after RNA interference and adjusted to shCTRL was performed. Significant differences were assessed using Student’s *t*-test (* *p* < 0.05). Error bars represent standard error at the mean (SEM).

From all of the small ncRNAs identified by the medium RNA-seq, 137 were considered intergenic since they were not embedded in known and yet annotated genes in hg19 or hg38 builds. Amongst these 137 intergenic sncRNAs, 49 were identified as snoRNAs using blast and dedicated software (see above). This observation raised the question of how these snoRNAs were transcribed since they were not embedded within a gene body. As above, we randomly selected 16 newly identified intergenic snoRNAs to assess their incorporation in snoRNPs and their localization in nucleoli. Among the 16 tested, 12 new intergenic snoRNAs can be considered genuine snoRNAs (Figure 4A,B).

Then, we hypothesized that these new intergenic snoRNAs could be in fact embedded in introns of yet unannotated transcripts, as evidenced by candidate #397 located in an intergenic region in hg19 but found to be sheltered within an intron of a longer transcript isoform of the *ATP11C* gene in the hg38 annotation. To assess if the levels of intergenic snoRNAs were dependent on splicing machinery, i.e., to test whether other intergenic snoRNAs could uncover introns of yet unannotated transcripts, we performed shRNA-mediated RNA interference against the general splicing factors U1 small nuclear ribonucleoprotein 70 kDa (SNRNP70), SNRNP40 (PRP8) and Splicing factor U2AF 65 kDa subunit (U2AF65) (Supplementary Figure S6). As shown in Figure 4C, the levels of validated intronic snoRNA candidates #16 and #80 (Figure 2 and Figure 3) used as positive controls were reduced by 74% and 24%, respectively, compared with that in control cells. The levels of intergenic snoRNA candidates #293 and #346 seemed to be unaffected by splicing inhibition (Figure 4C), suggesting that these candidates could represent autonomous snoRNAs transcribed from their own promoters such as SNORD3A, SNORD118 and SNORD13 [40]. Alternatively, the stability of these snoRNAs is higher than the kinetics of transient RNA interference, as shown in Supplementary Figure S7. In contrast, intergenic snoRNA candidates #300, #341, #358 and #397 showed between 20 and 48% decreases in their levels after the knockdown of the splicing machinery (Figure 4C). This is consistent with candidate #397 found a posteriori to be hosted in an intron of a newly annotated isoform of ATP11C. Hence, candidate #397 can be reclassified as a genuine intronic snoRNA whereas the other candidates await the identification of hosting transcripts.

**Figure 5:**
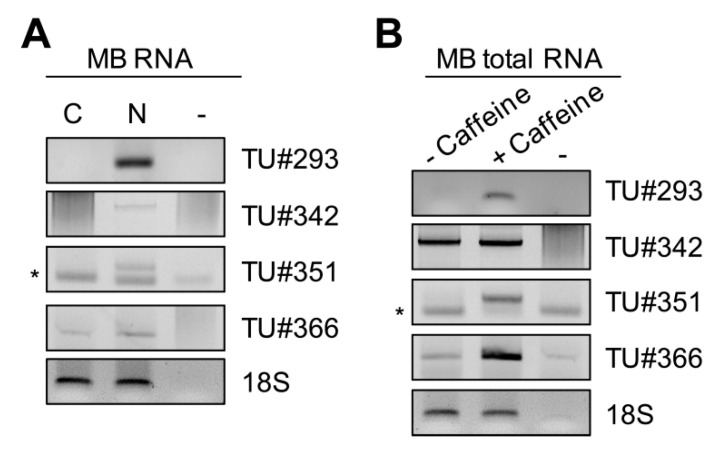
Discovery of new transcripts surrounding the new intergenic snoRNAs. (**A**) Nuclear and cytoplasmic fractionation of myoblasts (MBs) was performed prior to RNA extraction. Newly identified transcriptional units surrounding the intergenic snoRNA candidates (indicated as TU#) were detected by RT-PCR (n = 2). (**B**) MBs were treated with or without a 10nM solution of caffeine for 8 h to inhibit the non-sense mediated decay pathway. Then, total RNA from MBs was extracted, and new TU#s were detected by RT-PCR (n = 2); 18S ribosomal RNA (18S) was used as an invariant control; -: mock PCR; *: primer dimers.

We then took advantage of the total RNA-seq to investigate whether other intergenic snoRNAs could be hosted within introns of yet unannotated transcripts. We reconstructed transcriptional units (TUs) surrounding intergenic snoRNA candidates using STAR and Scallop as accurate reference-based transcript assemblers that feature high accuracy in assembling multi-exon transcripts as well as weakly expressed transcripts. From the results obtained, we reliably identified TUs and splicing junctions surrounding the candidates #293, #342, #351 and #366 (namely TU#293, TU#342, TU#351 and TU#366). As an experimental validation, we performed RT-PCR on MBs to detect the new TUs. However, we could only detect TU#342 from total RNA fractions (data not shown), suggesting that the candidate TUs might belong to the fraction of weakly expressed non-coding transcripts that preferentially locate in specific subcellular compartments. We therefore performed RT-PCR on RNAs isolated from cytoplasmic or nuclear extracts. Surprisingly, we detected PCR products of TU#293, TU#351 and TU#366 in nuclear but not in cytoplasmic fractions, consistent with our hypothesis (Figure 5A). TU#342, which was already detected in total RNA fractions, seemed to be enriched in the nucleus compared with in the cytoplasm (Figure 5A). Next, we assumed that, in addition to being part of weakly expressed transcripts restricted to the nucleus, some of the candidate SnoRNA Host Genes (SNHGs) may also be targeted by the nonsense-mediated decay (NMD) pathway [46]. To test this hypothesis, we inhibited the NMD with 10 mM of caffeine for 8 h [47] and tested expression of the newly identified TUs by RT-PCR. As shown in Figure 5B, we observed increased levels of all TUs after treatment with caffeine. Thus, these new transcripts seemed to be indeed sensitive to the NMD pathway.

Altogether, these findings suggest that the majority of newly identified intergenic snoRNAs can be reclassified as genuine snoRNAs (Figure 4). In addition, these intergenic snoRNAs are located between new potential exons (TUs) that can be part of yet unannotated transcripts, which are commonly called SNHGs. Being splicing-dependent (Figure 4C), we propose that these new intergenic snoRNAs are produced from introns of transcripts composed of newly identified TUs, which are mostly degraded by the NMD pathway (Figure 5).

## 3. Discussion

Here, we identified and experimentally validated new snoRNAs in the context of human muscle progenitors, which contributes to continuous improvement of the annotation of the human genome. Interestingly, half of the new snoRNAs were found in intergenic regions, which is quite remarkable since, at least in mammals, most snoRNAs are processed from the splicing of introns. We provided evidence that these intergenic snoRNAs are indeed hallmarks of the presence of not yet annotated transcription units. 

Previous studies aimed to systematically identify new small ncRNAs from yeast to human through plants, either on the basis of bioinformatics predictions to search for specific structures or motifs, or via specific sequencing methods dedicated to the identification of ncRNAs in a given size range (reviewed in [37]). For example, small RNA-seq approaches on RNAs smaller than 50 nt are dedicated to the identification of mature miRNAs and piRNAs, which usually excludes snoRNAs that are in a size range of 50–200 nt. To capture the snoRNAome in a given cellular context, other strategies have been designed. The TGIRT-seq is a structure-based approach dedicated to the identification of snoRNAs, with a slight bias towards highly structured SNORAs, whereas the PAR-CLIP is dedicated to the identification of ncRNAs co-precipitated with a core proteins of a given RNP [34,35], suggesting that they are indeed functional snoRNAs, with the limitation that certain snoRNAs belong to non-canonical snoRNPs (reviewed in [48]). Another major limitation of such bioinformatics or sequencing approaches is that they can also lead to false identification of non-functional genes or pseudogenes products that resemble snoRNAs [49]. Here, we proposed an unbiased sequencing approach with respect to structure, presence in RNP complexes and genomic location, which allowed us to capture the complete repertoire of small ncRNAs of more than 50 nt, to eliminate mature miRNAs in abundance and to capture sncRNAs of less than 200 nt independently of their host transcripts. We used a high sequencing depth (140 million reads) so as to not miss snoRNAs expressed at low levels. Although rRNA depletion prior to library construction may in turn deplete certain rRNA-associated snoRNAs, we verified that we retrieved the 402 already annotated snoRNAs and present in the DASHRv1 and snorna-lbme-db reference databases [50,51]; 15% of the pre-miRNAs, which is the normal fraction found in a given cell type [39]; and all tRNAs and snRNAs. The biocomputational prediction of the 400 new unannotated candidate sncRNAs did not show either a bias towards the identification of a specific type of sncRNAs. Surprisingly, 40% of the newly identified RNA sequences failed to be characterized as known ncRNA species by blast analysis or bioinformatics prediction software. Importantly, these are not degradation products since they were marked by well-defined peaks of reads. Hence, these transcripts could represent a new class of small ncRNAs that definitely need to be further investigated. 

We identified yet unannotated snoRNAs in the context of human muscle progenitors, either unique or belonging to already known families of snoRNAs but with distinct genomic locations. To fully experimentally validate these snoRNA candidates as genuine snoRNAs, we reasoned that they must fulfill certain requirements such as transcription of the host gene in the chosen context, their presence in snoRNP complexes and nucleolar localization. The host genes of the snoRNA candidates were indeed transcribed in the muscle context, as seen from the total RNA-seq, although there was no correlation between the levels of expression of the host gene and that of the corresponding snoRNA. This is also consistent with the number and the expression levels of the snoRNAs showing very little variation during muscle differentiation. This absence of correlation was already observed in numerous studies [36,52,53]. This suggests that the levels of snoRNAs are controlled at the posttranscriptional stage and not host gene transcripts abundance.

We then confirmed the incorporation of almost all snoRNA candidates into snoRNP complexes corroborating the functionality of the new snoRNAs. Of note, we only assessed interactions between snoRNAs and the snoRNP enzymes Dyskerin and Fibrillarin, although some snoRNAs interact with non-conventional protein partners to fulfill non-traditional functions [48,54]. For example, SNORD13 does not associate with Fibrillarin but can guide RNA acetylation by the RNA acetyltransferase NAT10 on the 18S rRNA ([55,56,57], reviewed in [58]). In addition, several SNORDs were found to be associated with heterogeneous nuclear RNP (*hnRNP),* RNA helicases or proteins involved in splicing rather than with the canonical methylating protein complex in order to regulate alternative splicing (reviewed in [54]). A few SNORAs and SNORDs associated with the protein kinase RNA-associated (PKR), leading to its activation following a metabolic stress or during muscle differentiation [59,60]. Hence, for the few snoRNAs for which we could not validate the association with classical core proteins of snoRNPs, non-canonical RNPs may be involved. Finally, we showed that the majority of the snoRNP candidates accumulated in nucleoli. Interestingly, we observed that two of them (candidates #26 and #80) were also located in the cytoplasm. Other examples exist such as a group of three snoRNAs (SNORD32A, 33 and 35A), which upon lipotoxic and oxidative stress, accumulate in the cytoplasm to participate in the propagation of cellular stress responses [61,62]. Thus, we can hypothesize that, in addition to guiding modifications in the nucleoli, the two cytoplasmic candidates #26 and #80 could operate unknown non-nucleolar functions in muscle cells such as exosome-mediated intercellular communication or degradation of RNAs [63]. In contrast, candidate #16, identified as a homolog of SNORA58, did not locate in nucleoli but accumulated in the nucleoplasm. It is likely that this candidate belongs to a subclass of H/ACA snoRNAs, called AluACA snoRNAs, which are processed and incorporated into H/ACA snoRNPs but are restricted to the nucleoplasm [64,65] with yet unknown function. Of note, SNORA58 also has an antisense element against SNORD32A. Therefore, its candidate homolog snoRNA #16 may also have a snoRNA as a target, which would then be modified in the nucleoplam [66]. Other research groups have also enriched the snoRNAome over the last few years. Interestingly, several newly identified snoRNAs were common to several studies including ours [34,35,66]. For instance, the candidate snoRNA #232, homologous to SNORA80D, was identified by PAR-CLIP [35] and was sequenced and included in the recent snoDB database [66]. In total, we uncovered and validated 18 new potential snoRNAs never identified by others. Interestingly, among these 18 snoRNAs, 17 did not share homology with any known snoRNAs or with each other. Importantly, these snoRNAs escaped identification in the previous studies [34,35,66], with one plausible explanation being that most of the identified snoRNA candidates showed levels of expression one log lower than the already known snoRNAs. However, we cannot exclude the possibility that the 18 new potential snoRNAs exhibited tissue-specific expression patterns [67]. Maybe the best explanation still remains that our method is dedicated to the identification of sncRNAs in the size-range of snoRNAs in contrast to classical RNA-seq that sequence long RNAs (>200 nt) or small RNA-seq that sequence short RNA (<50/80 nt).

Only a few examples of non-canonical intergenic snoRNAs with their own polymerase (Pol) II promoter have already been described, although they do not seem to function as genuine snoRNAs [40]. Rather than guiding the modification of RNAs, intergenic snoRNAs have been involved in other posttranscriptional processes, such as SNORD3A (U3), SNORD118 (U8) and SNORD13 (U13), which participate in the cleavage of pre-rRNAs and of the Telomerase RNA Component (TERC) [40,68]. However, up until now, no intergenic snoRNA guiding RNA modifications has been identified, at least in mammals. In 2010, by in silico prediction, the presence of Pol II promoters around snoRNAs located outside of host genes has been reported [69]. However, since then, no other study with experimental data has confirmed these predictions. Even if this possibility should not be entirely excluded, it seems more likely that these intergenic snoRNAs would be pseudogenes or retrogenes derived from snoRNA retrotransposition and are likely non-functional [40,49]. Here, we reported that almost all of the intergenic snoRNA candidates were embedded into a snoRNP complex and located in the nucleoli, therefore suggesting that they are genuine snoRNAs. One explanation for the misclassification of some snoRNAs as intergenic is likely due to the incomplete annotation of transcripts in the corresponding region [49]. Alternatively, it may be due to the known pervasive transcription of the genome [70], although at low levels, producing transcripts that are quickly degraded [71]. This hypothesis was reinforced by our findings of the splicing dependency of intergenic snoRNA candidates. In addition, we found the example of the intergenic candidate #397 that we reclassified as an intronic snoRNA originating from a transcriptional isoform of the *ATP11C* gene that emerged between the assembly of the hg19 and hg38 human genome builds. This led us to identify additional intergenic snoRNAs sheltered within what we called transcriptional units. Next, we confirmed that these transcriptional units were indeed subjected to degradation by the NMD pathway. These transcripts, referred to as SNHGs, are probably predominantly lncRNAs since all coding genes in the human genome are presumably annotated. Thus, we can conclude that the newly intergenic snoRNA candidates that we have identified are likely embedded in transcripts dedicated to the production of snoRNAs, whereas the fate of the host transcript itself is to be degraded by the NMD [10]. This type of mechanism has actually been documented as a way to regulate the expression of the host genes [36,46]. 

As a whole, we identified new sncRNAs and experimentally validated new unannotated snoRNA genes in the context of muscle cells. In addition, we provide evidence that new intergenic snoRNAs were in fact located within introns of new transcriptional units that can be used as hallmarks for the identification of yet unannotated SNHGs relevant for muscle function.

## 4. Materials and Methods

### 4.1. Cell Culture

We used myoblasts (MBs) and in vitro differentiated myotube (MT) counterpart cell lines [72]. Cells were grown as previously described [5,7].

### 4.2. RNA-Seq Protocols

For the total RNA-seq, RNAs were isolated as described below using TRI Reagent^®^, and subjected to Bioanalyzer. Only RNAs with a RIN > 8 were kept for further libraries construction and sequencing (GENOM’IC platform, Cochin Institute, France). Sequencing was performed with a 75-base-pair read length in a pair-ended manner.

For the medium RNA-seq, RNAs were first depleted in poly(A^+^) RNAs using three rounds of NEBNext Poly(A^+^) mRNA magnetic isolation module (New England Biolabs, Ipswich, MA, USA) according to the manufacturer’s instructions except that supernatants containing poly(A) depleted RNAs were recovered by phenol/chloroform extraction. Then, RNAs were depleted in rRNAs using the Ribo-Zero™ Magnetic Gold Kit (Illumina Inc., San Diego, CA, USA). RNAs wider than 200 nt were removed using NucleoSpin miRNA kit (Macherey-Nagel GmBH & Co. KG, Düren, Germany) and RNAs smaller than 50 nt were removed by gel purification using the methods described by Ellington and Pollard [73]. As stated before, only RNAs with a RIN > 8 were further processed. Libraries constructions were prepared using NEBNext Multiplex Small RNA Library Prep Set for Illumina (New England Biolabs, Ipswich, MA, USA). Gel purification at the end of the protocol was adapted to the wanted size of RNAs, i.e., between 50 and 200 nt (without the adaptors) or between 170 and 350 (with the adaptors). Sequencing was performed by Beckman-Coulter (Beckman Coulter, Brea, CA, USA) with a 125-base-pair read length in a pair-ended manner. 

Reads were trimmed and clipped using Trimmomatic (trimmomatic-0.33, with -phred33 illuminaclip:adapter.fa:2:30:10 leading:3 trailing:3 slidingwindow:4:15 minlen:50 as options). Alignments were performed using Star (Galaxy Version 2.7.8a) for the total RNA-seq and Bowtie2 for medium RNA-seq using hg19 from UCSC. 

The genomic data generated for this study were deposited in the GEO repository with the accession number GSE178649.

### 4.3. Antibodies

The primary antibodies used were directed against Dyskerin (DKC1, GTX109000, GeneTex, Irvine, CA, USA), Fibrillarin (FBL, GTX101807, GeneTex, Irvine, CA, USA), Coilin (COIL, GTX112570, GeneTex, Irvine, CA, USA), Lamin A/C (sc7292, Santa Cruz Biotechnology Inc., Dallas, TX, USA), α-Tubulin (sc32293, Santa Cruz Biotechnology), Nucleophosmin (NPM1 or B23, sc-55622, Santa Cruz Biotechnology Inc., Dallas, TX, USA), small nuclear Ribonucleoprotein 70 (snRNP70, AP17045-ev-AB, Abgent, San Diego, CA, USA), U2AF65 (AP14583a-ev-AB, Abgent), snRNP40 (PRP8, HPA026527, Atlas Antibodies, Bromma, Sweden) and irrelevant antibody (V5 Tag Monoclonal Antibody, R960-25, Thermo Fisher Scientific, Waltham, MA, USA).

### 4.4. RNA Preparation

Total RNA was isolated using Tri reagent^®^ (Sigma-Aldrich, Saint-Louis, MO, USA) according to the manufacturer’s instructions and as previously described [5,7]. Short and long RNAs were purified using Nucleospin^®^ miRNA (Macherey-Nagel GmBH & Co. KG, Düren, Germany) according to instructions [5].

### 4.5. RT-PCR

RNA was isolated as described above and reverse transcribed as described previously [5]. Primers used for amplification are described in Supplementary Table S4. Of note, primers were always chosen with the nucleotide in 3′ mapping a mismatch between the candidate snoRNA and its homolog when it exists to produce a “floating” 3′-end that prevents PCR amplification of the homolog but allows for amplification of the candidate to be tested. When this was not possible, primers were picked outside of the region of homology and complementary to the candidate snoRNA only. Amplification of the snoRNA candidates only was confirmed by in silico PCR tools from UCSC.

### 4.6. RNA and Protein Immunoprecipitation

The cells were lysed, and the RNA and proteins were extracted and quantified as described previously [5,7] and used for native RNA immunoprecipitation (RIP) or protein immunoprecipitation experiments, respectively. Nuclear protein extracts (10 mg) were incubated with the appropriate antibody (1 μg/mg of proteins) for 2 h at 4 °C, as described earlier [5,7]. Total, cytoplasmic and nuclear proteins were analyzed by SDS–PAGE and immunoblotting as previously described, or when appropriate, co-precipitated RNA was extracted using TRI Reagent method, reverse transcribed and PCR amplified (n ≥ 2), as described previously [5,7].

### 4.7. Nucleoli Isolation

Nucleoli isolation was performed as described previously [43] with minor modifications. Nuclei from myoblasts were isolated as described previously [5,7]. Pelleted nuclei were resuspended in 3 mL of Solution I (0.25 M sucrose, 10 mM MgCl_2_, Roche’s complete Protease Inhibitor Cocktail and Vanadyl ribonucleoside complexes (VRC, Sigma)), layered over 3 mL of Solution II (0.35 M sucrose, 0.5 mM MgCl_2_, Roche’s complete Protease Inhibitor Cocktail and VRC) and centrifuged at 1430× *g* for 5 min at 4 °C. Then, the nuclei pellet was resuspended in 3 mL of Solution II and sonicated in 10 s ON/10 s OFF six times in cold water using Diagenode Bioruptor UCD-200 at power setting “High”. The sonicated nuclei were checked under a phase contrast microscope to ensure that more than 90% of the nuclei were broken. Next, the sonicated solution was layered over 3 mL of Solution III (0.88 M sucrose and 0.5 mM MgCl_2_) and centrifuged at 2800× *g*. The supernatant corresponding to the nucleoplasmic fraction was removed and stored at −80 °C. The pellet containing the nucleoli was washed twice by resuspension in 500 µL of Solution II and centrifuged at 2000× *g* for 2 min at 4 °C. Finally, isolated nucleoli were resuspended in 500 µL of Solution II, transferred into a new tube and stored at −80 °C until further use. Total, cytoplasmic, nuclear, nucleoplasmic and nucleolar proteins were analyzed by SDS–PAGE and immunoblotting as previously described [5,7], or when appropriate, co-isolated RNA was extracted using TRI Reagent method, reverse transcribed and PCR amplified (n ≥ 2) as described previously [5,7].

### 4.8. Immunofluorescence Staining on Isolated Nucleoli

Purified nucleoli were spotted on poly-L-Lysine slides (Thermo Fisher Scientific, Waltham, MA, USA) and air dried. The slides were rehydrated with PBS for 5 min at room temperature (RT) and incubated with anti-NPM1 antibody (1:100) for 30 min at RT. Then, the slides were washed three times with PBS and incubated with goat anti-mouse IgG (Alexa fluor 488 conjugated; 1:250) for 30 min at RT. Finally, the slides were washed three times with PBS, counterstained with 0.66 mM Pyronin Y (Sigma-Aldrich, Saint-Louis, MO, USA) for 1 min, washed again three times with PBS and mounted with Vectashield.

### 4.9. Plasmids and Constructs

Short hairpin RNAs (shRNA) directed against human snRNP70, U2AF65, PRP8 and Luciferase were produced using MessageMuter™ shRNA production kit (Illumina Inc., San Diego, CA, USA) and in vitro transcribed using T7 RiboMax large-scale production system (Promega) following manufacturer’s instructions. Sequences are depicted in Supplementary Table S4.

### 4.10. Transfection Experiments

The knockdown of snRNP70 + U2AF65 was combined with that of the major splicing factor PRP8, since interference of snRNP70 or U2AF65 alone was insufficient to reduce splicing significantly in another study [74]. shRNAs were transiently transfected (n = 3) using the Lipofectamine RNAimax reagent (Thermo Fisher Scientific, Waltham, MA, USA), following the manufacturer’s instructions and as previously described [7].

### 4.11. Inhibition of the Transcription 

The myoblasts (MBs) were seeded the day prior the treatment to reach 70% confluency. Then, the MBs were treated with actinomycin D to inhibit the transcription by the RNA polymerase II (1 µg/mL). Total RNA was extracted at 0 h, 1 h, 4 h, 24 h and 48 h after the addition of the drug. Finally, the RNA levels of the candidates were assessed by RT-PCR, as described above.

### 4.12. Inhibition of the Non-Sense Mediated (NMD) Pathway

The myoblasts (MBs) were seeded at 70% confluency. Twenty-four hours later, the culture medium was replaced with a medium containing caffeine (10 mM) for 8 h. The total RNA from untreated and treated MBs was isolated as described above.

## Data Availability

The genomic data generated for this study were deposited in the GEO repository with the accession number GSE178649.

## Supplementary Materials

The following are available online at https://www.mdpi.com/article/10.3390/ncrna7030056/s1, Table S1: Small ncRNA candidates identified by medium RNA-seq; Table S2: Alignment/prediction of small ncRNA candidates; Table S3: Total; RNA-seq results expressed in CPM; Table S4: List of primers and oligonucleotides.
