# Supplementary material for: Systematic Identification and Functional Validation of New snoRNAs in Human Muscle Progenitors
Source: Noncoding RNA. 2021 Sep 13;7(3):56. doi: 10.3390/ncrna7030056 (PMC8482216; doi:10.3390/ncrna7030056)
Supplement: Supplementary file 1 [file ncrna-07-00056-s001.zip › Suppl_file.pdf]

Systematic identification and functional validation of new snoRNAs in human muscle progenitors

Baptiste Bogard[1,2], Claire Francastel[1,2,3], Florent Hubé[1,2,3]

[1] Université Paris Diderot, Sorbonne Paris Cité, Paris, France.

[2] Epigénétique et Destin Cellulaire, CNRS UMR 7216, Paris, France.

[3] Corresponding author:

Dr. Florent Hubé

Tel: +33 1 57 27 89 32

Fax: +33 1 57 27 89 10

E-mail: florent.hube@univ-paris-diderot.fr

[3] Corresponding author:

Dr. Claire Francastel

Tel: +33 1 57 27 89 18

Fax: +33 1 57 27 89 10

E-mail: claire.francastel@univ-paris-diderot.fr

UMR7216 - Epigénétique et Destin Cellulaire

Université Paris 7 Diderot

Bâtiment Lamarck - 4ème étage

Case Courrier 7042

35, rue Hélène Brion

75013 PARIS

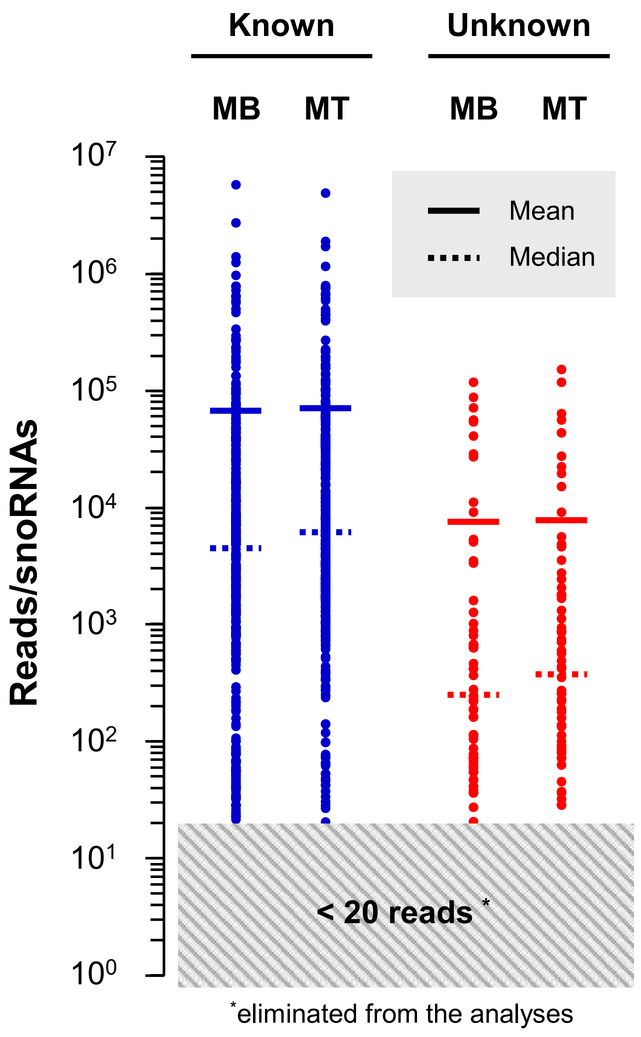

Supplemental Figure S1. Levels of expression between the known snoRNAs and the newly identified (unknown) snoRNA candidates. The graph shows that on average newly identified snoRNAs are 10 times less expressed than the known snoRNAs. MB, myoblasts; MT, myotubes.

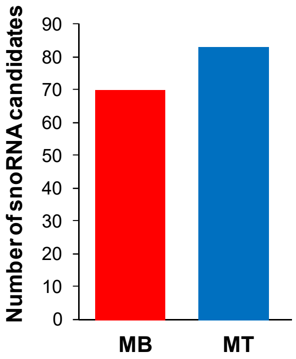

Supplemental Figure S2. Number of snoRNA candidates per cell origin. MB, myoblasts (red); MT, myotubes (blue).

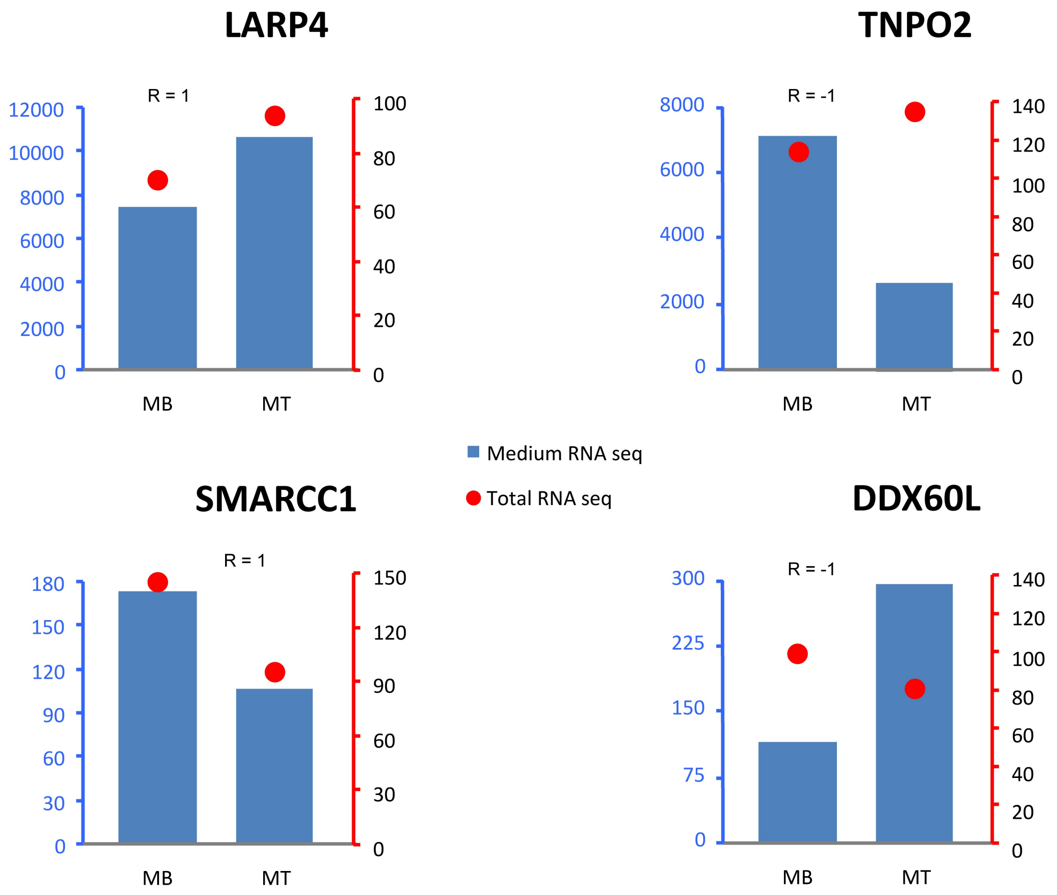

Supplemental Figure S3. Correlation between expression levels of snoRNA candidates and their respective host genes. Expression levels of snoRNA candidates are indicated with bars (blue) and expression levels of the host genes are indicated with dots (red). R represents the coefficient of correlation (pearson). LARP4, La Ribonucleoprotein 4; TNPO2, Transportin 2; SMARCC1, SWI/SNF Related, Matrix Associated, Actin Dependent Regulator Of Chromatin Subfamily C Member 1; DDX60L, DExD/H-Box 60 Like. MB, myoblasts; MT, myotubes.

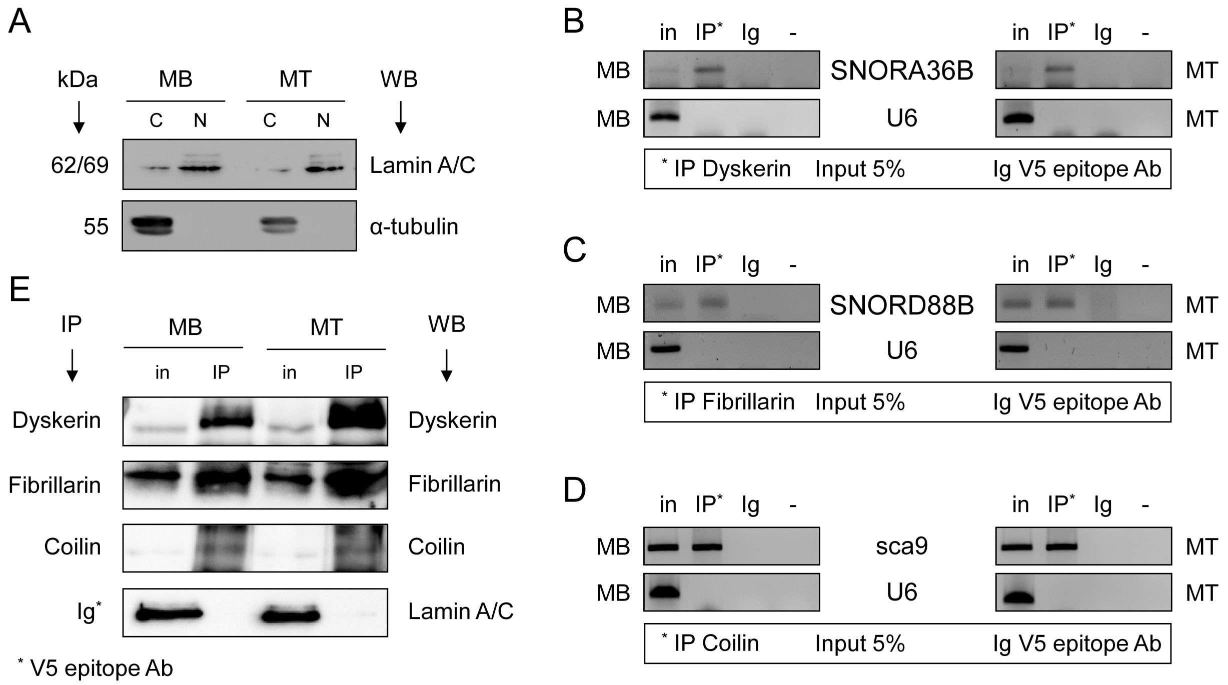

Supplemental Figure S4. Control of the efficiency of Immunoprecipitation IP-PCR experiments. (A) Immunoprecipitation were performed on nuclear extracts of myoblasts (MB) and myotubes (MT), controlled herein using Lamin A/C and α-tubulin antibodies as nuclear and cytoplasmic fraction, respectively. (B) IP-PCR against Dyskerin. (C) IP-PCR against Fibrillarin. (D) IP-PCR against Coilin. Known SNORA36B, SNORD88B and scaRNA9 (sca9) were used as positive control, U6 snRNA (U6) as negative control. (E) Native IP was performed using the indicated antibodies and the effective protein precipitation was confirmed by western blotting. kDa, kiloDalton; C, cytoplasmic fraction; N, nuclear fraction; IP, immunoprecipitation; WB, western blot; in, 5% input; Ab. Antibody; -, mock PCR.

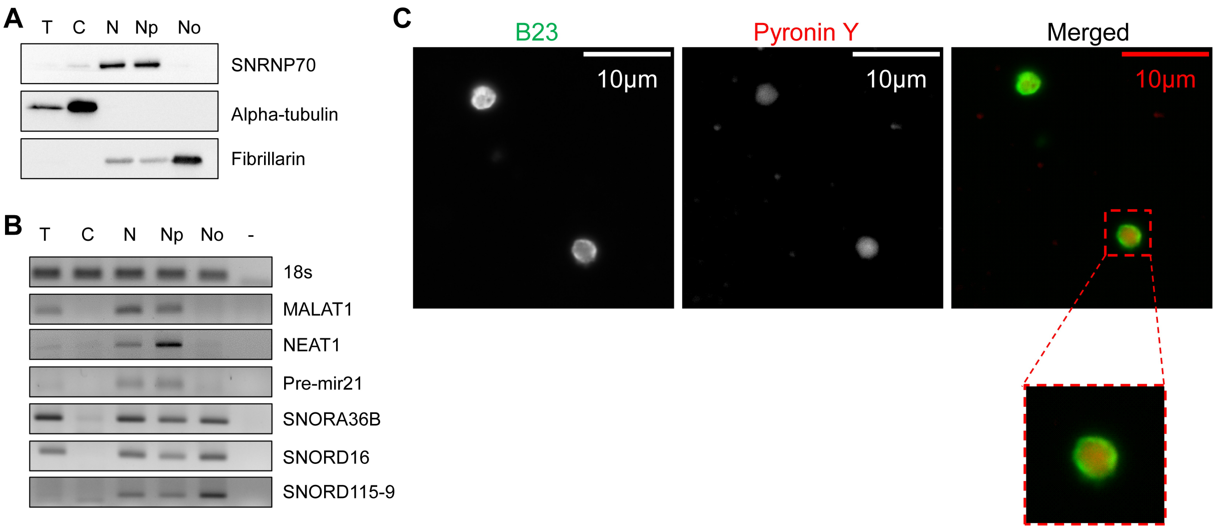

Supplemental Figure S5. Control of the purity of nucleoli. (**A**) Isolation of nucleoli by sucrose cushion centrifugation was performed as described in Material and Methods section and the efficiency of the purification was confirmed by western blotting. SNRNP 70 was used as a nuclear marker. Alpha-tubulin was used as a cytoplasmic marker. Fibrillarin was used as nucleolar marker. (**B**) Total RNA from each fractions was extracted as described in Material and Methods section to assess the correct localization of known ncRNAs by RT-PCR (n=2). 18s rRNA was used as positive control for each fraction. NEAT1 RNA, MALAT1 RNA and pre-miRNA-21 (pre-mir21) were used as a nuclear marker. Known SNORNA36B, SNORD16 and SNORD115-9 were used as a nucleolar marker. T, total fraction; C, cytoplasmic fraction; N, nuclear fraction; Np, nucleoplasmic fraction; No, nucleolar fraction; -, mock PCR. (**C**) Isolated nucleoli from myoblasts were immunostained with anti-B23 (green) antibody and stained Pyronin Y (red) to confirm their integrity.

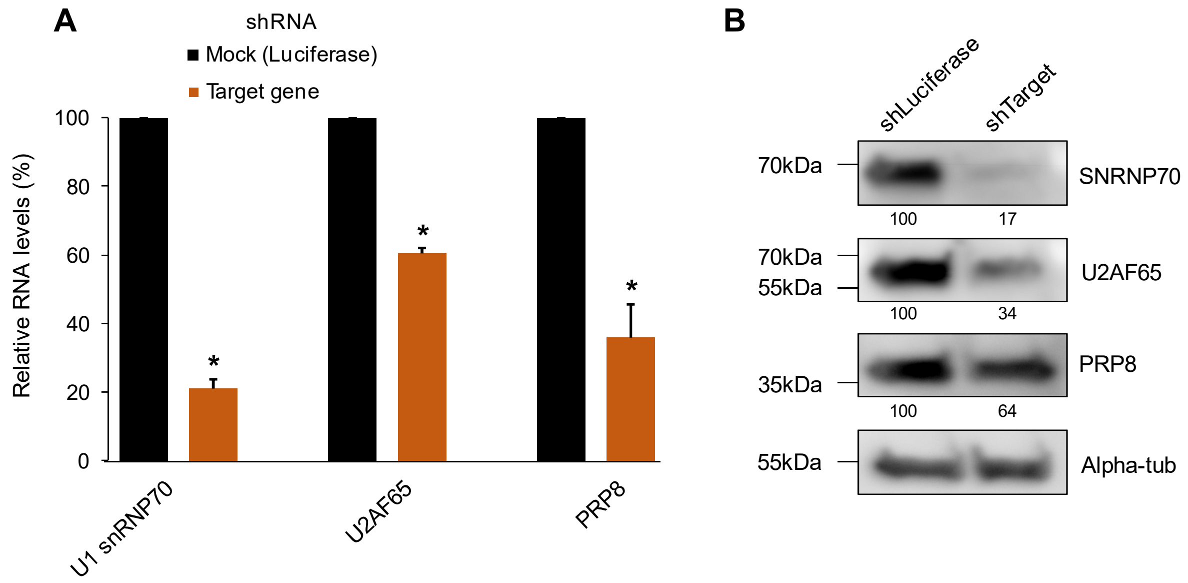

Supplemental Figure S6. Control of the knockdown efficiency. Knockdown experiments were performed as described in Material et Methods section. The efficiency of the knockdown was assessed by (A) RT-qPCR and (B) Western blot. A quantification of the remaining protein levels after RNA interference and adjusted to the mock control is given below each corresponding panel. U1, snRNP70; U2, U2AF65; alpha-Tub, alpha-Tubulin. Significant differences were assessed using Student's t-test (*$P < 0.05$). Error bars represent standard error at the mean (SEM).

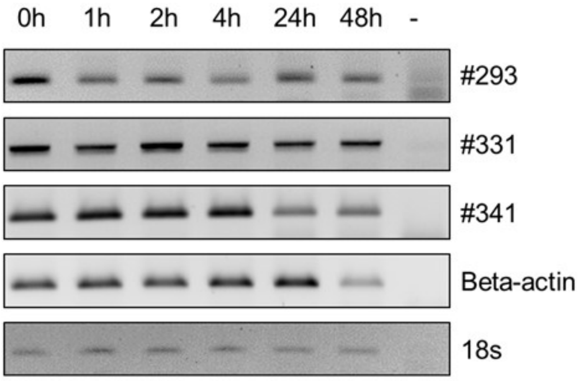

Supplemental Figure S7. Stability of new intergenic snoRNA candidates. The transcription in myoblasts was inhibited using actinomycin D (1µg/mL). Total RNA was extracted at the time points indicated in the figure after the addition of the drug. RNA levels of the candidates (indicated by #) were analyzed by RT-PCR. Ribosomal RNA 18s (18s) was used as an invariant control due to its high stability. -: mock PCR. Candidates were amplified by a 35 cycles PCR while beta-actin and 18s were amplified in a 25 cycles PCR.
