# Supplementary material for: Systematic Identification and Functional Validation of New snoRNAs in Human Muscle Progenitors
Source: Noncoding RNA. 2021 Sep 13;7(3):56. doi: 10.3390/ncrna7030056 (PMC8482216; doi:10.3390/ncrna7030056)
Supplement: Supplementary file 1 [file ncrna-07-00056-s001.zip › TableS2.pdf]

Query= identified_RefSeq ID database used (**+NCBI**)
Sbjct= candidate as used in Table S1 Software(s) used
Experimental validation Host gene of the candidate

Alignment/sequence identified

H/ACA or C/D boxes as defined in database(s) or identified by software(s)

Comments

Query= SCARNA21_NR_003000.1 Length = 138 snoRNA-LBME-db
Sbjct= #4 Length = 146 Blast
IP COIL DNAJC16

```
Query: 1   ggctcgatttcctggggggtggtctcagcccactccacctcccctcagccgagcctagag 60
           |||||| ||||||||||||||| ||||| | || |||||||||||||||| |||||| ||
Sbjct: 8   ggctcggtttcctggggggtgggctcagtctacaccacctcccctcagccaagcctacag 67

Query: 61  tagaggggccaggcatcctccc-caggggaggggcgttgaagcaaggagcctctcctggg 119
           ||||||| ||||||||||||||  | ||||| ||||||  || |||||||||| ||||||
Sbjct: 68  tagagggcccaggcatcctcccgtatgggagaggcgtttgagtaaggagcctcacctggg 127

Query: 120 ctgtcctagcctcacattt 138
            ||||||||||||||||||
Sbjct: 128 gtgtcctagcctcacattt 146
```

Query= SNHG3_HG491818.1 Length = 185
Sbjct= #8 Length = 206 Blast
IP DYSK, FIB, COIL and Nucleolar localization SNHG3

```
Query: 99  tttgcttatcagctctttgtcaatgatttctgtaatggaaataggattgaagagactttt 158
           ||||||||||||||||||||||||||||||||||||||||||||||||||||||||||||
Sbjct: 1   tttgcttatcagctctttgtcaatgatttctgtaatggaaataggattgaagagactttt 60

Query: 159 attctagttggtcaggatttacctctg 185
           |||||||||||||||||||||||||||
Sbjct: 61  attctagttggtcaggatttacctctg 87
```

no box defined yet

Query= SNORA73B_NR_004404.2 Length = 205 snoSeeker
Sbjct= #9 Length = 296 Blast
SNHG3

```
Query: 1   tccaacgtggataccctgggaggtcactctccccaggctctgtccaagtggcataggga 60
           ||||||||||||||||||||||||||||||||||||||||||||||||||||||||||||
Sbjct: 25  tccaacgtggataccctgggaggtcactctccccaggctctgtccaagtggcataggga 84

Query: 61  gcttagggctctgccccatgatgtacagtccctttccacaacgttgaagatgaagctggg 120
           ||||||||||||||||||||||||||||||||||||||||||||||||||||||||||||
Sbjct: 85  gcttagggctctgccccatgatgtacagtccctttccacaacgttgaagatgaagctggg 144

Query: 121 cctcgtgtctgcgcctgcatattcctacagcttcccagagtcctgtggacaatgactggg 180
           ||||||||||||||||||||||||||||||||||||||||||||||||||||||||||||
Sbjct: 145 cctcgtgtctgcgcctgcatattcctacagcttcccagagtcctgtggacaatgactggg 204

Query: 181 gagacaaaccatgcaggaaacatat 205
           |||||||||||||||||||||||||
Sbjct: 205 gagacaaaccatgcaggaaacatat 229
```

Query= SNORA63C_NR_145725.1 Length = 131 snoRNA-LBME-db
Sbjct= #12 Length = 231 Blast

```
                                                OSCP1

Query: 1   aagcaggattcagactacaatatagctgttaagtgctgtattgtcattccccctgctcaa 60
           ||||||||||||||||||||||||||||||||||||||||||||||||||||||||||||
Sbjct: 211 aagcaggattcagactacaatatagctgttaagtgctgtattgtcattccccctgctcaa 152

Query: 61  attaaagttgtttcttaactataccccatctgctattctgtagcagccagggatgcttggt 120
           ||||||||||||||||||||||||||||||||||||||||||||||||||||||||||||
Sbjct: 151 attaaagttgtttcttaactataccccatctgctattctgtagcagccagggatgcttggt 92

Query: 121 cacatacatgt 131
           |||||||||||
Sbjct: 91  cacatacatgt 81

----------------------------------
Query= SNORD145_NR_145816.1     Length = 100     snoSeeker
Sbjct= #13                     Length = 108     Blast
                                                TMEM53

Query: 1   accagggcctgggagggatgatgtaccaagtgagctgaatggaatcacttggcagcactg 60
           ||||||||||||||||||||||||||||||||||||||||||||||||||||||||||||
Sbjct: 105 accagggcctgggagggatgatgtaccaagtgagctgaatggaatcacttggcagcactg 46

Query: 61  taggcccaagccagagctgctctgacatcccaggcccagg 100
           ||||||||||||||||||||||||||||||||||||||||
Sbjct: 45  taggcccaagccagagctgctctgacatcccaggcccagg 6

----------------------------------
Query= SNORD160_NR_145793.1     Length = 73      snoSeeker
Sbjct= #14                     Length = 108     Blast
                                                KIF2C

Query: 1  gggcagtgatgagaggggaggaggctctggggcctcagggcctactgtatactcctctgt 60
          ||||||||||||||||||||||||||||||||||||||||||||||||||||||||||||
Sbjct: 22 gggcagtgatgagaggggaggaggctctggggcctcagggcctactgtatactcctctgt 81

Query: 61 gactctgaacctg 73
          |||||||||||||
Sbjct: 82 gactctgaacctg 94

----------------------------------
Query= SNORA26_NR_003016.1      Length = 122     snoRNA-LBME-db
Sbjct= #15                     Length = 141     Blast
                                                OSBPL9

Query: 1   gtgcccttttaaggttgacccagtgctttaagaggctaacacagaagggtaaagtaagtc 60
           ||||||||||||||||||| ||||||||||| ||||||||||||||||||||| |||||
Sbjct: 129 gtgcccttttaaggttgacgcagtgctttaaggggctaacacagaagggtaaaggaagtc 70

Query: 61  tccataaaacccagagaagagactggaaagctcctctttggatcctgtctggagtcaca 119
           |||||||| ||||||||||||| || ||||||||||||| || ||| ||||||||||||
Sbjct: 69  tccataaaccccagagaagagattgtaaagctcctctttagaacctttctggagtcaca 11

----------------------------------
Query= SNORA58_NR_002985.2      Length = 236     snoRNA-LBME-db
Sbjct= #16                     Length = 140     Blast
IP DYSK                                         NDC1

Query: 1   gggcatactcgtagaccttgcctgactgtgctcatgtccaggcagggggacagtgtatg 60
           |||||||| |||||||||||||||||||||||||||||||||||||||||||| |||||
Sbjct: 139 gggcatacccgtagaccttgcctgactgtgctcatgtccaggcagggggacattgtatt 80

Query: 61  caagaataatttggagttcctgccagctctaaccagcttcatcagtggctggataaattg 120
           | ||| ||||||| |||||||||||||| || ||||||| |||||||||||||||||| |
```

```
Sbjct: 79  cgagattaatttgaagttcctgccagctttatccagcttaatcagtggctggataaatag 20

Query: 121 caggact 127
            |||||||
Sbjct: 19  caggact 13

---------------------------------
```

Query= SNORA44_NR_002976.1 Length = 132 snoRNA-LBME-db
Sbjct= #23 Length = 100 Blast
CGN

```
Query: 33  gccatgggatctccaactgcatgcaagagcaacctggaaagactttgacagcgcaggtca 92
            |||||||||||||||||||||||||||||||||||||| ||||||||||||  |||||||
Sbjct: 93  gccatgggatctccaactgcatgcaagagcaacctggagagactttgacagaacaggtca 34

Query: 93  gtacaatacctgcaagctgccact 116
            | ||||| ||||| ||||||||||
Sbjct: 33  gcacaatccctgc-agctgccact 11

---------------------------------
```

Query= SNORD64_NR_001294.1 Length = 67 snoSeeker
Sbjct= #25 Length = 67 Blast
C1orf204

```
Query: 1  ggatttgtgatgagctgtgtttactgagcatgatgaagtaaagctcaacgtgattactct 60
           |||||| | ||||||||||||||||||||||| ||||||||||| ||||  ||||||||||
Sbjct: 67 ggatttataatgagctgtgtttactgagcattatgaagtaaagttcaatatgattactct 8

Query: 61 gaagtc 66
           ||||||
Sbjct: 7  gaagtc 2

---------------------------------
```

Query= SNORA67_NR_002912.1 Length = 137 snoRNA-LBME-db
Sbjct= #26 Length = 145 SnoReport/Blast
IP DYSK and Nucleolar localization ABL2

```
Query: 1   atccaaggtgattccctctccaagggga-catc-agtgcc--tctcaggaaagtagcagc 56
            |||||||||||||||||||||||||||| |||| ||||||  ||||||||||||||| |
Sbjct: 2   atccaaggtgattccctctccaaggggaacatccagtgcccctctcaggaaagtagcaac 61

Query: 57  ttggaatagaatctggcatgcctaaggcctttggggaactgggatgcttatttcctctgc 116
             ||||| |||||||||||||||||||| |||||||||| ||| || |||||||||||||
Sbjct: 62  ctggaacagaatctggcatgcctaaggcatttggggaacagggctggttatttcctctgc 121

Query: 117 cttccttggctgcccacatgg 137
            |||||||||||||| ||||||
Sbjct: 122 cttccttggctgcctacatgg 142

---------------------------------
```

Query= SNORA72_NR_002581.1 Length = 132 snoRNA-LBME-db
Sbjct= #28 Length = 135 Blast
NUCKS1

```
Query: 7   atattctcgctgttctgattttgtaatagtcaggacaggctaaacattcgctatattaag 66
            |||||||| ||||| |||||||||||||||||||||||||||| ||||| || |||||||
Sbjct: 128 atattctcactgttttgattttgtaatagtcaggacaggctaaccattcacttta ttaag 69

Query: 67  accatgcatgtgtccccaaacctagttctttccctaggtctggtttcataaatgctggtg 126
            ||||||||| | |||||| | ||||||||||||  |||||| |||| ||||||| || ||
Sbjct: 68  accatgcatatatccccagaactagttctttcctgaggtctagttttataaatgttgctg 9

Query: 127 ataaac 132
            ||||||
```

```
Sbjct: 8   ataaac 3
```

Query= SNORA26_NR_003016.1 Length = 122 snoRNA-LBME-db
Sbjct= #31 Length = 79 Blast
INTS7

```
Query: 45   aagggtaaagtaagtctccataaaacccagagaagagactggaaagctcctctttggatc 104
            |||| ||||||||||||| | |||||||||||||||||||||||| ||||||| ||||||
Sbjct: 1    aaggataaagtaagtctctacaaaacccagagaagagactggaaaactcctctctggatc 60

Query: 105  ctgtctggagtcaca 119
            ||| |||||||||||
Sbjct: 61   ctgcctggagtcaca 75
```

Query= SNORD116_NR_003330.2 Length = 94 snoSeeker
Sbjct= #32 Length = 66 Blast
USH2A

```
Query: 9   tgatgacttccatatatacattccttggaaagctgaacaaaatgagtgaaaactcta 65
           ||||||||||||||||| |||||||||||||||||||||| ||||||||||||||||
Sbjct: 60  tgatgacttccatatatccattccttggaaagctgaacaacatgagtgaaaactcta 4
```

Query= SNORD142_NR_132758.2 Length = 195 snoSeeker
Sbjct= #34 Length = 194 SnoReport/Blast
PFKP

```
Query: 5    ctgcaatgatgatgaaatagtaggcatatgtgtgatcttcttaaagaatccacaacggtt 64
            ||||||||||||||||||||||||||||||||||||||||||||||||||||||||||||
Sbjct: 1    ctgcaatgatgatgaaatagtaggcatatgtgtgatcttcttaaagaatccacaacggtt 60

Query: 65   atcagattaaagctgtgacccatccccagctcagatgagttcatgaaatcctgatttaag 124
            ||||||||||||||||||||||||||||||||||||||||||||||||||||||||||||
Sbjct: 61   atcagattaaagctgtgacccatccccagctcagatgagttcatgaaatcctgatttaag 120

Query: 125  attttaaaataatgaagattgttagttgacttgttcggtttattaaaatcctcatcttac 184
            ||||||||||||||||||||||||||||||||||||||||||||||||||||||||||||
Sbjct: 121  attttaaaataatgaagattgttagttgacttgttcggtttattaaaatcctcatcttac 180

Query: 185  tgagcgggggg 195
            |||||||||||
Sbjct: 181  tgagcgggggg 191
```

Sbjct= #36 Length = 165 SnoReport
FRMD4

```
Hpos=52 ACApos=126 37 162 AAAGCA 86 ATA 160 0.71 H/ACA box snoRNA
CAAAAAACATTACAACATTTTGTAATTTTTCTAACTTCAAGTATTTTTTGGAGGTTTGTTGTAGAACTATGGAAACTTGA
AACATAAAGCAAGACTATTTAAAGGTTCAATCTCGTCTTGTAAGAAAGAGAAACAGGTAGAGAGACAGAAAGGGGATATA
TACTT
```

Sbjct= #38 Length = 129 SnoReport
IP FIB and Nucleolar localization KIAA1217

```
C=11 D=89 23 125 ATGATGG 33 CTGA 111 0.96 C/D box snoRNA
TGATTCATTTTCTTCTACCCACCCCCCAAACAATGATGGAGTTGCAAGGGTCTGAGCACAACTGCACCAGCAGAAAGGGA
TATGTCTATACATGTCCCTAAGCGGCTACTCTGAGCTTTGGGATGTGTG
```

Sbjct= #41 Length = 158 SnoReport

IP COIL PLAU

C=11 D=72 10 95 CTGATGA 20 CTGA 81 0.99 C/D box snoRNA
Hpos=45 ACApos=109 30 138 ACAGGA 72 ACA 136 0.95 H/ACA box snoRNA
GGTGAGCATCACTGACCTGCTGATGACAGTGGGGTGGAAGGGGACAAACTTACATGTCCCCTTATTCCATCACAGGAGGA
CTGAGGAGGTGGGGGTGCCCGAGAGGGATGCTTTCTCCTACCTGCCTCCCTAAGACATCCCTCTGTTTGTCCTCCAG

Query= SNORD172_NR_145812.1 Length = 59 snoSeeker
Sbjct= #43 Length = 78 SnoReport/Blast
KAT6B

```
Query: 1  ggttcctgagatgatgcagctggaatgtgcttggctggctggctgtcctgatcagaacc 59
           |||||||||||||||||||||||||||||||||||||||||||||||||||||||||||
Sbjct: 3  ggttcctgagatgatgcagctggaatgtgcttggctggctggctgtcctgatcagaacc 61
```

Query= SNORA7B_NR_002992.2 Length = 139 snoRNA-LBME-db
Sbjct= #45 Length = 139 Blast
STIM1

```
Query: 3   cctcctgggatcgcatctggagactgcctagtattctgccagcttcggaaagggagggaa 62
            |||||||||||| |||||||| | ||| |||||||||||| |||||  ||||||||||||
Sbjct: 3   cctcctgggatcccatctggaaagtgcttagtattctgcctgcttcacaaagggagggaa 62

Query: 63  agcaagcctggcagaggcacccattccattcccagcttgctccgtagctggtgattggaa 122
            ||||| | |||  |||||||| ||||||| ||||||||||| |||||||||||||||||
Sbjct: 63  agcaaacgtgg-tgaggcaccctttccatt-ccagcttgctcggtagctggtgattggaa 120

Query: 123 gacactctgcgacag 137
            ||||||||| |||||
Sbjct: 121 gacactctgtgacag 135
```

Query= SNORD133_NR_132753.1 Length = 216 snoSeeker
Sbjct= #54 Length = 270 Blast
LARP4

```
Query: 1  cagcccagtgatgatcactattcctacttagagagaataggactaactttcagaaatcca 60
          ||||||||||||||||||||||||||||||||||||||||||||||||||||||||||||
Sbjct: 30 cagcccagtgatgatcactattcctacttagagagaataggactaactttcagaaatcca 89

Query: 61 ggca 64
          ||||
Sbjct: 90 ggca 93

Query: 155 gcctggatcgagattgttaagtccctctcagtgaagggtaagattatgagatctgagggc 214
            ||||||||||||||||||||||||||||||||||||||||||||||||||||||||||||
Sbjct: 184 gcctggatcgagattgttaagtccctctcagtgaagggtaagattatgagatctgagggc 243

Query: 215 tg 216
            ||
Sbjct: 244 tg 245
```

Sbjct= #57 Length = 71 SnoReport
IP FIB and Nucleolar localization HCFC2

C=11 D=54 5 72 ATGATGT 15 CTGA 58 0.95 C/D box snoRNA
GCTTCAGGGATTTGATGATGTTTACGAGCCAAGGACCAAAACCTACTGACAAAGAATCTGAAAACCCTGAA

Query= SNORA9_NR_145743.1 Length = 131 snoRNA-LBME-db
Sbjct= #63 Length = 153 SnoReport/Blast

```
Query: 1   gcaagcctccagcgtgcttgggtctgcagtgaccccgtggattcctacagggcttgccag 60
           ||||||||||||||||||||||||||||||||||||||||||||||||||||||||||||
Sbjct: 16  gcaagcctccagcgtgcttgggtctgcagtgaccccgtggattcctacagggcttgccag 75

Query: 61  aacagttttgaaatggtttgaggccttgccgtgctccatgtagagcaaggttatagaaat 120
           ||||||||||||||||||||||||||||||||||||||||||||||||||||||||||||
Sbjct: 76  aacagttttgaaatggtttgaggccttgccgtgctccatgtagagcaaggttatagaaat 135

Query: 121 ttcagacaatg 131
           |||||||||||
Sbjct: 136 ttcagacaatg 146
```

Sbjct= #64 Length = 120 SnoReport

ULK1

C=11 D=58 22 93 ATGATGA 32 CTGA 79 0.71 C/D box snoRNA
CATGAGTAGGGTTGCAGGGTGATGGCCCCACATGATGATGGCACTCCTTGTGTGACATGGGAGGGGTCTGCAGAGCACCT
GAGTCGGCCATGAACCCCCGTCCCTCTGTCCATCTGACTG

Query= SNORA101B_NR_135620.1 Length = 132 snoSeeker

Sbjct= #68 Length = 197 Blast

RALGAPA1

```
Query: 1   ttctttttatatgcccttctgatcccaagtatggcagaaggatataaaggtcaccaagaa 60
           ||||||||||||||||||||||||||||||||||||||||||||||||||||||||||||
Sbjct: 164 ttctttttatatgcccttctgatcccaagtatggcagaaggatataaaggtcaccaagaa 105

Query: 61  taatttggaggttgccaaggacttatttaaaagtgttttcaagtccgaccgagaactctg 120
           ||||||||||||||||||||||||||||||||||||||||||||||||||||||||||||
Sbjct: 104 taatttggaggttgccaaggacttatttaaaagtgttttcaagtccgaccgagaactctg 45

Query: 121 acatttacaaat 132
           ||||||||||||
Sbjct: 44  acatttacaaat 33
```

Query= SNORD58B_NR_002572.1 Length = 66 snoRNA-LBME-db

Sbjct= #69 Length = 81 Blast

PRPF39

```
Query: 5  gatgatggcatttcttaggacaccttggattaataatgaaaacaactactctctgagca 64
          ||||||||||||||||||||||  ||||||||||| ||||||||||||||||||||||||
Sbjct: 11 gatgatggcatttcttaggacaattttggattaattatgaaaacaactactctctgagca 70
```

Query= SNORD115-21_NR_003313.1 Length = 81 snoSeeker

Sbjct= #75 Length = 147 SnoReport/Blast

SNORD115-15

```
Query: 1   gggtcgatgatgagaaccttatattttctgaagagaggtgatgacttaaaaatcatgctc 60
           ||||||||||||||||||||||||||||||||||||||||||||||||||||||||||||
Sbjct: 22  gggtcgatgatgagaaccttatattttctgaagagaggtgatgacttaaaaatcatgctc 81

Query: 61  aataggattacgctgaggccc 81
           |||||||||||||||||||||
Sbjct: 82  aataggattacgctgaggccc 102
```

Sbjct= #80 Length = 170 SnoReport

IP DYSK and Nucleolar localization THBS1

```
Hpos=50 ACApos=110 3 112 AAAACA 50 ACA 110 0.93 H/ACA box snoRNA
GGTGAGTAGCGAGTTCTTAGATCCTAAGAGACTGATGCATACATGGGGAAAAACAAATATAAAACCTGGCAGTTGTACCT
ATCCCTGTGGGTGCTGAGGATGTCTAGGAACATGATGGAGAACCTTCTGAAGGCTGCAGGTTTTAACCTGGCTCTGGGCT
CTTCTTCCAG

----------------------------------
Query= SNORA24_NR_145768.1      Length = 131     snoRNA-LBME-db
Sbjct= #82                      Length = 185     Blast
                                                 PARP16

Query: 1    ctccatgtatctttggacctgtcaagtgtggcagtctcccttccttgccatggaagagc 60
            ||||||||||||||||||||||||||||||||||||||||||||||||||||||||||||
Sbjct: 160  ctccatgtatctttggacctgtcaagtgtggcagtctcccttccttgccatggaagagc 101

Query: 61   atattcttgtttaccagcaaagctgtcaccatttaattggtatcagattctgacttgcac 120
            ||||||||||||||||||||||||||||||||||||||||||||||||||||||||||||
Sbjct: 100  atattcttgtttaccagcaaagctgtcaccatttaattggtatcagattctgacttgcac 41

Query: 121  aagtaacattc 131
            |||||||||||
Sbjct: 40   aagtaacattc 30

----------------------------------
Query= SNORD148_NR_145840.1     Length = 54      snoSeeker
Sbjct= #89                      Length = 70      Blast
                                                 GPT2

Query: 1   ggcagtcagattcctcaaggcaaagacagtgcccttctgtcctcatcatcctca 54
           ||||||||||||||||||||||||||||||||||||||||||||||||||||||
Sbjct: 54  ggcagtcagattcctcaaggcaaagacagtgcccttctgtcctcatcatcctca 1

----------------------------------
Query= SNORA31_NR_002967.1      Length = 130     snoRNA-LBME-db
Sbjct= #93                      Length = 81      Blast
                                                 ALDH3A2

Query: 52   actaggatgcaaaagaaagaaatccctgcgctttctgtctgtctttgtggcggcccagat 111
            ||||||||||||||||||  |||||| || || |||| ||| |||| ||||| ||||||||
Sbjct: 3    actaggatgcaaaagaaaacaatccttgtgccttctatctctcttggtggcagcccagat 62

Query: 112  tgaattggggaatacatct 130
            |||||||||||||||||||
Sbjct: 63   tgaattggggaatacatct 81

----------------------------------
Sbjct= #107                     Length = 204     SnoReport
                                                 VMP1

Hpos=59 ACApos=129 5 133 AGAGTA 61 ACA 131 0.95 H/ACA box snoRNA
CTAGTGGTGATAAATGTGGGACTTCTGAGAAGTCATTCATTTTATTCTTTGTGCCATACCAGAGTACAGTATCAGCTGAG
CTGACCTTACTCTGAGGACTAACTCTTTTGCTGGAAGCGGTTTCTGATTTACAGCTCTTGGTTTCTCCCAGACATGTTGG
TGGGAGAGATTTTGGTTTTTAAGGGGTTGTTAGATGGAGTAAAT

----------------------------------
Query= SNORD134_NR_132755.1     Length = 181     snoSeeker
Sbjct= #112                     Length = 191     Blast
                                                 FASN

Query: 1    cccccgtgtgatgagcacacagctgcagcatagcaagaacccaggggctctggtgaagcc 60
            ||||||||||||||||||||||||||||||||||||||||||||||||||||||||||||
Sbjct: 189  cccccgtgtgatgagcacacagctgcagcatagcaagaacccaggggctctggtgaagcc 130

Query: 61   aacacacgggtcatcagggttccctcagactggtgccctctgttggcacccatctctgag 120
            ||||||||||||||||||||||||||||||||||||||||||||||||||||||||||||
```

```
Sbjct: 129 aacacacgggtcatcagggttccctcagactggtgccctctgttggcacccatctctgag 70

Query: 121 ccccatgggccacatgtgggcaggacctggaaagaaccttggctgcagaggactgagggg 180
           ||||||||||||||||||||||||||||||||||||||||||||||||||||||||||||
Sbjct: 69  ccccatgggccacatgtgggcaggacctggaaagaaccttggctgcagaggactgagggg 10

Query: 181 g 181
           |
Sbjct: 9   g 9
```

Query= SNORA81_NR_002989.1 Length = 178 snoRNA-LBME-db

Sbjct= #114 Length = 126 Blast

MIB1

```
Query: 3   cttcttgtataagcactgtgctaaaattgcagacactaggaccatgtcttggtttttgca 62
           |||| |||||||||||||||||||||| ||||| ||||||||||| ||||||||||||  
Sbjct: 123 cttcctgtataagcactgtgctaaaatcgcagaaactaggaccatatcttggtttttgtg 64

Query: 63  ataatgcttgcagagtacacacaagaagaaaagtaacagcactagattgtaaagactggg 122
           |||||||| |||||||||||| ||||| ||| ||||||||| | |||| |||||||| ||
Sbjct: 63  ataatgctagcagagtacacagaagaataaacgtaacagcattggattataaagactagg 4

Query: 123 gtg 125
           |||
Sbjct: 3   gtg 1
```

Sbjct= #118 Length = 76 SnoReport

NFIC

C=11 D=50 7 70 TTGATGT 17 CTGA 56 0.68 C/D box snoRNA
AGCGTTCATGTTCACGTTGATGTTCATAGTCACGTACATGTTCAGACGGCTGCAGCTGAGTGAGCATGGCCGGGGT

Query= SNORD135_NR_132756.1 Length = 75 snoSeeker

Sbjct= #122 Length = 131 Blast

TNPO2

```
Query: 1   gcctcaagtgatggagagcaatacccggggatgactgtgaccacattgggatgtattcgt 60
           ||||||||||||||||||||||||||||||||||||||||||||||||||||||||||||
Sbjct: 111 gcctcaagtgatggagagcaatacccggggatgactgtgaccacattgggatgtattcgt 52

Query: 61  actgtctgatggggc 75
           |||||||||||||||
Sbjct: 51  actgtctgatggggc 37
```

Query= SNORA68B_NR_145718.1 Length = 133 snoSeeker

Sbjct= #124 Length = 144 Blast

ANKRD27

```
Query: 1   ttctcacctaaacccaagaatcactgtttcttatagcggtggtttaaacagaggtgcaaa 60
           ||||||||||||||||||||||||||||||||||||||||||||||||||||||||||||
Sbjct: 141 ttctcacctaaacccaagaatcactgtttcttatagcggtggtttaaacagaggtgcaaa 82

Query: 61  cagcaagtgaatctcgtcgcctttgcggggctgtggccatgcccctcaaaggaaatttgg 120
           ||||||||||||||||||||||||||||||||||||||||||||||||||||||||||||
Sbjct: 81  cagcaagtgaatctcgtcgcctttgcggggctgtggccatgcccctcaaaggaaatttgg 22

Query: 121 aggctctacagcc 133
           |||||||||||||
Sbjct: 21  aggctctacagcc 9
```

```
----------------------------------
Query= SNORA2A_NR_002950.1      Length = 135     snoRNA-LBME-db
Sbjct= #127                     Length = 136     Blast
                                                 C2orf48

Query: 1   taggccctgaatcaagaccaatggtttgctgtagctgttggtttcaaacaggagctaaga 60
           |||||||||||||||||| | || ||||||||||||||||||||||||||||| |  |||
Sbjct: 135 taggccctgaatcaagactagtgttttgctgtagctgttggtttcaaacaggatccgaga 76

Query: 61  gtgatgtcttccttgtggtctgttggctattcagtattccagtgcgaattgccaattcag 120
           ||||||||||||||||||||||||  ||  ||||||| ||||||  | | ||||||||||
Sbjct: 75  gtgatgtcttccttgtggtctgttcactgctcagtatcccagtgtcagtggccaattcag 16

Query: 121 ttggaagaaacat 133
           ||||||| |||||
Sbjct: 15  ttggaagcaacat 3

----------------------------------
Sbjct= #130                     Length = 100     SnoReport
                                                 DYSF

C=11 D=61 14 88 ATGAGGA 24 CTGA 74 0.90 C/D box snoRNA
AAAGAGAAAGGCCAAAGTGAGCCATGAGGATGCCTTTGTGAATGCCAGAGGACATTACCTCTGTTGTTTGGAACTGAGCT
CTCTCTCCACCTAGGTGGTT

----------------------------------
Query= SNORA40B_NR_145723.1     Length = 128     snoRNA-LBME-db
Sbjct= #135                     Length = 192     SnoReport/Blast
                                                 RAB3GAP1

Query: 1   tgcacttatgtatgtttttgtttaacttgtggacaaagacttatggataggtgcaaaaaa 60
           ||||||||||||||||||||||||||||||||||||||||||||||||||||||||||||
Sbjct: 37  tgcacttatgtatgtttttgtttaacttgtggacaaagacttatggataggtgcaaaaaa 96

Query: 61  taaatcctcttttgcaacccagaactcattgttcagtatgagttttgatacatataagaa 120
           ||||||||||||||||||||||||||||||||||||||||||||||||||||||||||||
Sbjct: 97  taaatcctcttttgcaacccagaactcattgttcagtatgagttttgatacatataagaa 156

Query: 121 gggatatt 128
           ||||||||
Sbjct: 157 gggatatt 164

----------------------------------
Query= SCARNA16_NR_003013.1     Length = 187     snoRNA-LBME-db
Sbjct= #138                     Length = 182     Blast
IP COIL                                          CCDC150

Query: 4   gtctggggaaaggctcctgtgttgttgagcctgccaagttgtgggtgcagcatggtacca 63
           ||||| ||||||||| |||||||||||| | |||| || ||||||||||||   ||||||
Sbjct: 179 gtctgtggaaaggctactgtgttgttgaacttgcccagctgtgggtgcagccatgtacca 120

Query: 64  ggcagcccaaccctgacctaaagtaaattcccggaagaagtctttcctggttttgaatt 123
           |||||||||| |||||| ||||||||||||||| | ||||||||||||||||||||||||
Sbjct: 119 ggcagcccaagcctgacataaagtaaattcccaggagaagtctttcctggttttgaatt 60

Query: 124 tgcagtaacaggtgtgagcattctagcagcagtttgatgatcatgtatgatactgcaaaca 183
           ||||||||||||||| ||||||| |||||||||||| |||||||||   ||   |   ||
Sbjct: 59  tgcagtaacaggtgtaagcattccagcagcagtttgttgatcatgttggaagcaacatgg 1

----------------------------------
Query= SNORA26_NR_003016.1      Length = 122     snoRNA-LBME-db
Sbjct= #144                     Length = 79      Blast
                                                 PCNA
```

```
Query: 4  cccttttaaggttgacccagtgctttaagaggctaacacagaagggtaaag-taagtctc 62
          ||||||||||| |||||||||||||||||||||| | |||||||||||||| ||||||||
Sbjct: 65 cccttttaaggctgacccagtgctttaagaggctgatacagaagggtaaagttaagtctc 6

Query: 63 ca 64
          ||
Sbjct: 5  ca 4
```

Partial sequence; lack of ACA

```
-----------------------------------
Query= SNORD83A_NR_000028.1     Length = 75      snoRNA-LBME-db
Sbjct= #160                     Length = 215     Blast
                                                 RPL3

Query  1    gctgttcagtgatgaggcctggaatgtgcgctgggcacagcgcccgagacagactgcgga  60
            ||||||||||||||||||||||||||||||||||||||||||||||||||||||||||||
Sbjct  181  gctgttcagtgatgaggcctggaatgtgcgctgggcacagcgcccgagacagactgcgga  122

Query  61   accgttccttgttgccttccttctgagaacagc  93
            |||||||||||||||||||||||||||||||||
Sbjct  121  accgttccttgttgccttccttctgagaacagc  89
```

Partial sequence; no box found

```
-----------------------------------
Query= SNORD83A_NR_000027.1     Length = 95      snoSeeker
Sbjct= #161                     Length = 159     Blast
                                                 RPL3

Query: 1   gctgttcgttgatgaggctcagagtgagcgctgggtacagcgcccgaatcggacagtgta 60
           ||||||||||||||||||||||||||||||||||||||||||||||||||||||||||||
Sbjct: 129 gctgttcgttgatgaggctcagagtgagcgctgggtacagcgcccgaatcggacagtgta 70

Query: 61  gaaccattctctactgccttccttctgagaacagc 95
           |||||||||||||||||||||||||||||||||||
Sbjct: 69  gaaccattctctactgccttccttctgagaacagc 35

-----------------------------------
Query= SNORD146_NR_145791.1     Length = 130     snoSeeker
Sbjct= #166                     Length = 142     Blast
                                                 SMARCC1

Query: 1   tcttatttcaaggctctgtgatgtgtaggagaaaatgtagaaccatagaatttcattatt 60
           ||||||||||||||||||||||||||||||||||||||||||||||||||||||||||||
Sbjct: 137 tcttatttcaaggctctgtgatgtgtaggagaaaatgtagaaccatagaatttcattatt 78

Query: 61  attactatttttgtctcatttagtcctctcattttctggtatagagggactgagagcc 120
           ||||||||||||||||||||||||||||||||||||||||||||||||||||||||||||
Sbjct: 77  attactatttttgtctcatttagtcctctcattttctggtatagagggactgagagcc 18

Query: 121 tgaagaggtt 130
           ||||||||||
Sbjct: 17  tgaagaggtt 8

-----------------------------------
Query= SNORD136_NR_132757.1     Length = 147     snoSeeker
Sbjct= #168                     Length = 197     SnoReport/Blast
                                                 GNL3

Query: 1   tgtgagaaatgatgagggtcaacattcttcataccaaagtgaagacatgagatccaactc 60
           ||||||||||||||||||||||||||||||||||||||||||||||||||||||||||||
Sbjct: 20  tgtgagaaatgatgagggtcaacattcttcataccaaagtgaagacatgagatccaactc 79

Query: 61  tgagctcaccctgttgctaaatggataatgccagtactctcttgtggaaggtattaccag 120
           ||||||||||||||||||||||||||||||||||||||||||||||||||||||||||||
```

```
Sbjct: 80   tgagctcaccctgttgctaaatggataatgccagtactctcttgtggaaggtattaccag 139

Query: 121  aacaagggatgtagttctgatcatttt 147
            |||||||||||||||||||||||||||
Sbjct: 140  aacaagggatgtagttctgatcatttt 166
```

Sbjct= #171 Length = 390 SnoReport
GPR128

```
Hpos=59 ACApos=130 51 180 ACAGGA 107 ACA 178 0.83 H/ACA box snoRNA
CAGTTCTTCCGTATTTCTTATTTCAAATATCTTGGTTAGTTCAGCTGTCTGGACTAGCTTATTCTTACTGCCAGCATATA
TCATTTGTTGTTCAGGCTTACATCCAACAGGACTGGAGAAAATAAAGCACGGAGGATATGGAACTCTCCCATCCATTATG
TTGATATTTATAACTATACACAATGAAACGAGGTTGTCGTTCAGGTAGTTCATCTTTAAGTTCATCTGGTGAAATACCCT
CAAGCTCCTCATCCAGTACCACCAGGCGTTTATCCTTCAATCTTCATTACAATAGAAGCATTGTTTGTTTCTTTGTGAAA
ACGAAACTTTCTCAGCTTTTCCACTAAATCTTCAGCAACATCAAAACCACCAAAGACTCACTCATTTCCT
```

Query= SNORA5A_NR_002919.1 Length = 134 snoRNA-LBME-db
Sbjct= #172 Length = 132 SnoReport/Blast
MYLK

```
Query: 1    tgcagccgtgtcaaattcagtacctgtcctatgcatggtaggcactggcccagaaggctg  60
            |||||| ||||||||||  || |||| |||   |   |||||||||| ||||||||||||
Sbjct: 1    tgcagctgtgtcaaatttggtgcctgccct-gcctgtgtaggcactgacccagaaggctg  59

Query: 61   ccacagaaacactgtgactcatgggccctgttcctgtgtcccaggctcagggataaattt 120
            ||||||||  ||  ||||||||||||||||||||||||||| | |||||||||||||||||
Sbjct: 60   ccacagaa--acactgactcatgggccctgttcctgtgtctcgggctcagggataaattt 117

Query: 121  ggttacagacatca- 135
            ||||||||| | ||
Sbjct: 118  ggttacagataccaa 132
```

Sbjct= #173 Length = 213 SnoReport
AMOTL2

```
Hpos=83 ACApos=156 11 166 AGACAA 91 ACA 164 0.57 H/ACA box snoRNA
GAAACCCCAGCTCAGCTCAGGGCCCCATGGGTGGGAGCCTCCTCTGTCAAGAGCCATCATCCCTGAGGGGATCCTAGGCC
CAGTACATAGAGACAAGCATCTCTCTATCAGGGCAGCTCCAAAGGGGTCTCAGCCTGGCTCTCTGAGTTCATCTGCACTT
TTGACAGAGAATAGAGATTCCCAATTTTTCTCTAGAACAAAAGAAGTAAGTCC
```

Query= SNORA18_NR_002959.1 Length = 132 snoRNA-LBME-db
Sbjct= #175 Length = 138 Blast
LINC00578

```
Query: 2    ttgaggtctatcccgatggggcttttcctgtagcctgcacatcgttggaaacgcctcata 61
            ||||||||| |||||||||| |||||| ||||| ||||||||| ||||||||||||||||
Sbjct: 133  ttgaggtctttcccgatgggacttttc-tgtaggctgcacatcattggaaacgcctcata 75

Query: 62   gagt-aactctgtggtttactttactcacaggactattgttagatctgtgggaa 115
            |||| ||||||||||||||||||||||||||||||| ||||||||||||||||||
Sbjct: 74   gagttaactctgtggtttactttactcacaggactcttgttagatctgtgggaa 20
```

Partial sequence; lack of ACA

Query= SNORA63_NR_033236.1 Length = 134 snoRNA-LBME-db
Sbjct= #176 Length = 284 Blast
LINC00888

```
Query: 1    gaagcaggattcagactacaatatagctgtttagtgctgtgttgtcgttcccccctgctca  60
                  |  |      ||||||||||||||  |||||| ||||||| || ||||
```

```
Sbjct: 126 aggttggattcattgttacaatatagctgtgaagtgctatgttgtccttgccccctgctc 185

Query: 61  aagtaaggttgtttcttactatacctgtctg-ctctatccctgtagcagccagggacgct 120
            ||   |  | |||||  || ||  ||||||  |||  | |       || || |||||||
Sbjct: 186 aaagcaatt-gtttcccacaatctctgtctcactcggttctatagtaagacatggacgct 244

Query: 121 tggtctcatacaagc 135
            |||||||| |||
Sbjct: 245 tggtctcaaacatta 259
```

Query= SNORA63_NR_033236.1 Length = 134 snoRNA-LBME-db

Sbjct= #177 Length = 169 SnoReport/Blast

LINC00888

```
Query: 1   gaagcaggattcagactacaatatagctgtttagtgctgtgttgtcgttcccccc-tgctc  59
              ||   |  ||    |||| | |||||||  |||||||  ||||| || |||| |||||
Sbjct: 25  tgaggcaggatctagttacattgtagctgtgaagtgctgcattgtctttgccccctgctc  84

Query: 60  aaagtaaggttg-tttcttactatacctgtctgctctatccctgtagcagccagggacgc 119
            ||| |||   || |  ||| | |  |||||||||  |    ||||||||||  |||| ||
Sbjct: 85  aaaataaaactgttacctttcaagccctgtctgccatggtgctgtagcagca-gggatgt 143

Query: 120 ttggtctcatacaagc 134
            ||||||||||||| |
Sbjct: 144 ttggtctcatacatgt 159
```

Query= SNORA63B_NR_145766.1 Length = 125 snoSeeker

Sbjct= #178 Length = 89 Blast

EIF4A2

```
Query  48   cctgcttaaagcacttgatgcataactctgtctaccttcattccgtagtaagacagagac  107
            ||||||||||||||||||||||||||||||||||||||||||||||||||||||||||||
Sbjct  1    cctgcttaaagcacttgatgcataactctgtctaccttcattccgtagtaagacagagac  60

Query  108  gcttggcttcagacattt  125
            ||||||||||||||||||
Sbjct  61   gcttggcttcagacattt  78
```

Query= SNORA48_NR_002918.1 Length = 135 snoRNA-LBME-db

Sbjct= #179 Length = 117 SnoReport/Blast

TMED11P

```
Query: 47  catatcagccagggacaaagcaactccttgttcatcccagcttggcttttgatccgtgcc 106
            ||||| |||||||||||||||||| | ||||||||||||||||||||||||||| || ||
Sbjct: 28  catattagccagggacaaagcaaccctttgttcatcccagcttggcttttgatctgtacc 87

Query: 107 catgcctggttcatgccttggacacat 133
             || ||||||||||||||| |||||||
Sbjct: 88  tatacctggttcatgccttagacacat 114
```

Query= SNORA62_NR_002324.1 Length = 154 snoRNA-LBME-db

Sbjct= #181 Length = 126 Blast

GNRHR

```
Query: 33  actgactggccgatgaactcgcaagtgtaggtagtgtgctacatgagggcaagttttc- 91
            |||||||||| |||||| || |||| ||||||||| ||||||||||||||  ||||||
Sbjct: 2   actgactggctgatgaaatcccaagcgtaggtagtatgctacatgaggggtgagttttt 61

Query: 92  gctaacaccacaagggtctctggcccaatgagtggagtttgatagtaattcttgctacaa 145
            ||||||||||||| ||||||||||||||||||||||||||||||| | ||||||  ||||
```

```
Sbjct: 62  gctaacaccacaaaggtctctggcccaatgagtggagtttgataggagttcttgtgacaa 115

----------------------------------
Query= SNORA51_NR_002981.1      Length = 132     snoSeeker
Sbjct= #186                    Length = 137     Blast
                                                 DDX60L

Query: 7   ctggtgcttaccacaggctgtgttcttacactgactgtatagaaagaggaggtagagtaa 66
           ||||||||||||||||||||  |||||||||||||| |||||||| ||||||||||||||
Sbjct: 7   ctggtgcttaccacaggctgaattcttacactgactatatagaaa-aggaggtagagtaa 65

Query: 67  acctaccccatatacacctcagctcaggccctgtgcctggtctgtattgtgaatgggga 126
           |||||||| |||||| ||||||| ||||| ||||||||| ||| ||||||||||| ||||
Sbjct: 66  acctacccaatataccccctcagcccaggctctgtgcctgatctatattgtgaatgtggga 125

Query: 127 acatag 132
           ||||||
Sbjct: 126 acatag 131

----------------------------------
Sbjct= #189                    Length = 116     SnoReport
                                                 AHRR

C=11 D=75 1 89 GGGATGA 11 CTGA 75 0.72 C/D box snoRNA
TGTCTGCTGAGGGATGAGGCACTGTTGGAGGTCCTGGGACAGGGGCCTGACCTCCTCAGCCTCTTCTGTGGCTTCTGACA
GCAGCAGTGGTAATACCGGTAACACATAGTGGTGAT

----------------------------------
Sbjct= #190                    Length = 135     SnoReport
IP FIB and Nucleolar localization               TRIO

C=11 D=79 27 119 ATGATGA 37 CTGA 105 0.88 C/D box snoRNA
CTAATTACTCAGATCAGCCAAGAAGGGTCCAGGATCATGATGAGCCGTGCTCAGGTTCACGTGGTGGTTTTGAAGCTGGG
CTAGACTTGGAAGTCTGTCGGACACTGAGAATCCATGGAATTTATGAAGACCAGAT

----------------------------------
Query= SNORD170_NR_145815.1     Length = 85      snoSeeker
Sbjct= #191                    Length = 100     Blast
                                                 TRIO

Query: 1  ggggcctgcaatgatggctgtgagctgcaggactgagcacaggttggagtcctggagttt 60
          ||||||||||||||||||||||||||||||||||||||||||||||||||||||||||||
Sbjct: 7  ggggcctgcaatgatggctgtgagctgcaggactgagcacaggttggagtcctggagttt 66

Query: 61 tgtgtgcatctgaggttcaggctcc 85
          |||||||||||||||||||||||||
Sbjct: 67 tgtgtgcatctgaggttcaggctcc 91

----------------------------------
Query= SNORA40_NR_002973.1      Length = 127     snoRNA-LBME-db
Sbjct= #195                    Length = 127     Blast
IP DYSK and Nucleolar localization              FAM196A

Query: 8   atgtatgtttttgtttaacgtgga--caaagacttacagataggtgcaaaaaataaatcc 65
           |||||||||||||||||| || |   ||||| ||||  |||||||||||||| ||||| |
Sbjct: 121 atgtatgtttttgtttaacttgtatacaaaggcttatggataggtgcaaaaa-taaattc 63

Query: 66  tctttgcaacccagaactcattgttcagtatgagttttgatacatataagaagggatat 125
           |||||||||||||||||||||||||||| ||||||||||||||||||||||||||||||
Sbjct: 62  tctttgcaacccagaactcattgttcagtgtgagttttgatacatataagaagggatat 3

Query: 126 ta 127
           ||
Sbjct: 2   ta 1
```

Query= SNORA109_NR_132964.1 Length = 128 snoRNA-LBME-db
Sbjct= #198 Length = 517 Blast
IP DYSK and Nucleolar localization DHFR

```
Query: 14   aaaatatactatgaaagtggctttaatacc-ctgaagacacaatagctaagacccaaact 72
            |||||| |||| ||||||||||||||  |  ||||| ||||||||||||||||||||||||
Sbjct: 88   aaaataaactacgaaagtggctttaacgcatctgaacacacaatagctaagacccaaact 29

Query: 73   gggattagataccccactatgcttagcc 100
            ||||||||||||||||||||||||||||
Sbjct: 28   gggattagataccccactatgcttagcc 1
```

Partial sequence; lack of ACA

Query= SNORA40_NR_002973.1 Length = 127 snoRNA-LBME-db
Sbjct= #220 Length = 136 SnoReport/Blast
FKPB5

```
Query: 13   tgttttgtttaac--gtggacaaagacttacagataggtgcaaaaaataaatcctcttt 70
            |||||||||||||  |||||||||||||||  | |||||||||||||||||||||||||
Sbjct: 18   tgttttgtttaacttgtggacaaagacttatggttaggtgcaaaaaataaatcctcttt 77

Query: 71   tgcaacccagaactcattgttcagtatgagttttgatacatataagaaggtgttgcagg 129
            |||||||||||||||||||||||||||||||||||||||||||||||||| |||
Sbjct: 78   tgcaacccagaactcattgttcagtatgagttttgatacatataagaagggatatta   134
```

Query= SNORD45B_NR_002748.1 Length = 71 snoRNA-LBME-db
Sbjct= #221 Length = 76 Blast
IP FIB and Nucleolar localization BTBD9

```
Query: 1   ggtcaatgatgtaatggcatgtattagctgaatctaaagttgatgtgagttctagaatta 60
           |||||||||||||||||||||||||||||||||| | ||||||| ||||||||| |||||
Sbjct: 2   ggtcaatgatgtaatggcatgtattagctgaatccagagttgatatgagttctaaaatta 61

Query: 61 cactga 66
          ||||||
Sbjct: 62 cactga 67
```

Query= SNORA8_NR_002920.1 Length = 139 snoRNA-LBME-db
Sbjct= #222 Length = 129 Blast
USP49

```
Query: 14   atctgcactcagcagtttacacctgctagggtgttcaaaggtcagtgctatagaaattca 73
            |||||||||||| ||||||  |||||| |||||| ||||| ||||||| |||||||||||
Sbjct: 1    atctgcactcagtagtttattcctgct-gggtgtccaaagatcagtgccatagaaattca 59

Query: 74   gtatctggcatcgttggttttcttggctttgtgcttgtt-aaacctggtatttctactga 132
            |||||| |||||  || |||||||||||||||||||||| |||| ||||||||||| |||
Sbjct: 60   gtatctagcatcactgattttcttggctttgtgcttgttaaaacttggtatttctattga 119

Query: 133 tacagta 139
             ||||||
Sbjct: 120 cacagta 126
```

Query= SCARNA15_NR_003011.1 Length = 127 snoRNA-LBME-db
Sbjct= #224 Length = 128 Blast
IP COIL and Nucleolar localization XPO5

```
Query: 5    agactaagaaaatagagtccttgaaatcaagctgactctgcttttagcctcctaaatgaa 64
```

```
           |||||||||||||||||||||| || ||||||||| |||||| |||||||||||||||||
Sbjct: 6   agactaagaaaatagagtccttaaagtcaagctgagtctgctgttagcctcctaaatgaa 65

Query: 65  aaggtagatagaacaggtcttgtttgcaaaataaattcaagacctacttatctaccaaca 124
           | | |||| ||||||||||||||||||||||||||||||||||||||||||||||| |||
Sbjct: 66  aggatagacagaacaggtcttgtttgcaaaataaattcaagacctacttatctaccgaca 125

Query: 125 gca 127
           |||
Sbjct: 126 gca 128

-----------------------------------
Query= SNORA40_NR_002973.1      Length = 127     snoRNA-LBME-db
Sbjct= #227                    Length = 159     SnoReport/Blast
IP DYSK and Nucleolar localization              AMD1

Query: 1   tgcacttatgtatgtttttgtttaac--gtggacaaagacttacagataggtgcaaaaaa 58
           ||||||||||||||||||||||||||  ||||||||||||||   || ||||||||||||
Sbjct: 4   tgcacttatgtatgtttttgtttaacttgtggacaaagactttaggaaaggtgcaaaaaa 63

Query: 59  taaatcctcttttgcaacccagaactcattgttcagtatgagtttgatacatataagaa 118
           |||||| |||||||||||||||||||||||||||||||||||||||||||||| ||||
Sbjct: 64  taaatcttcttttgcaacccagaactcattgttcagtatgagtttgatacatatcagaa 123

Query: 119 gggata 124
            |||||
Sbjct: 124 tggata 129

-----------------------------------
Query= SNORA27_NR_002575.1      Length = 126     snoRNA-LBME-db
Sbjct= #228                    Length = 117     Blast
                                                PEX7

Query: 1   tacccccttttcactttgccagttggacttatgtctttattggtcattcaagtgggg 57
                   || ||||||||||  ||||  |||||||||| ||||||||| |||||||
Sbjct: 117         ttccactttgccaactggatgtatgtctttactggtcattccagtgggg 62

Query: 58  caaaggaaatatcc-tttaaaactcaggcaaactgggtgtttgtctgtatcctgtc 103
           |||| ||||||||| |||||||||||||||||||||||||||||||    || |||
Sbjct: 61  caaacgaaatatcctttttaaaactcaggcaaactgggtgtttgtcatggtcatgta 13

Query: 58  agaggaaacaaat 103
               |  | |
Sbjct: 12  tcctgtcagag   1

-----------------------------------
Query= SNORA116_NR_145798.1     Length = 147     snoSeeker
Sbjct= #231                    Length = 158     Blast
                                                TULP4

Query: 2   ttttctcagtggtgcaagaagattaagccacattctggctttagagaggcatttctgaga 61
           ||||||||||||||||||||||||||||||||||||||||||||||||||||||||||||
Sbjct: 1   ttttctcagtggtgcaagaagattaagccacattctggctttagagaggcatttctgaga 60

Query: 62  gagatgaaggacacttcgttccccagccccaacctaagcatgtgactgtactcaccttgt 121
           ||||||||||||||||||||||||||||||||||||||||||||||||||||||||||||
Sbjct: 61  gagatgaaggacacttcgttccccagccccaacctaagcatgtgactgtactcaccttgt 120

Query: 122 cagatgctgttggaacctggctgaca 147
           ||||||||||||||||||||||||||
Sbjct: 121 cagatgctgttggaacctggctgaca 146

-----------------------------------
Query= SNORA80B_NR_028374.1     Length = 136     snoRNA-LBME-db
```

Sbjct= #232 Length = 185 Blast
AIMP2

```
Query: 1   gcatgggtttggatttatgacaggcccgtcaccctgggcctgtcatagtaccccatgcca 60
           ||||||||||||||| |||||||||||| || |||||| ||| ||||||| ||||||||||
Sbjct: 23  gcatgggtttggatt-atgacaggcccatccccctggacctctcatagtgccccatgcca 81

Query: 61  gagcaaactgtgtccccgaaccattgcctggcctctgtgcccgtaggctgctggcactga 120
           |||||||||||| ||||||||||||||||||| ||||| ||||| |||  ||||||||||
Sbjct: 82  gagcaaactgtggccccgaaccattgcctggcttctgtacccgtgggccactggcactga 141

Query: 121 agtgggttgcacagtg 136
           || ||||| |||||||
Sbjct: 142 agagggttacacagtg 157
```

Query= SNORA20_NR_002960.1 Length = 132 snoRNA-LBME-db
Sbjct= #233 Length = 150 Blast
POU6F2

```
Query: 1   cttcccatttatttgctgcttgtagtctcacagtgatacgagcagttatacgcatgggat 60
           ||||||||||||||||||||| ||||||||| |||||| ||||| |||||| ||||||||
Sbjct: 147 cttcccatttatttgctgcttatagtctcactgtgatatgagcaattatacacatgggat 88

Query: 61  aaaataacattgggccactgtaaattgagatgaagtaaccattttcatctcttctgcagg 120
           ||||||| |||| ||||||||||||||||||||||||||| ||||||| ||||||||| |
Sbjct: 87  aaaataatattgagccactgtaaattgagatgaagtaaccgttttcatatcttctgcatg 28

Query: 121 gactagacattg 132
           ||||||||||||
Sbjct: 27  gactagacattg 16
```

Query= SNORA15_NR_002957.1 Length = 133 snoRNA-LBME-db
Sbjct= #235 Length = 205 Blast
CCT6P3

```
Query: 1   gcatggccgaatactgtgtt--tttatcagtagtttacacagccagacaccatgcaaaag 58
           ||||||| ||||||||||||  ||||||||||||||||||||||||||||||||||||||
Sbjct: 4   gcatggctgaatactgtgttcttttatcagtagtttacacagccagacaccatgcaaaag 63

Query: 59  cagtcttccctttagaatgactgatggtatgctaaggtttttcatagcatatcattatta 118
           ||||||||||||||||||||||||||||||||||||||||||||||||||||||||||||
Sbjct: 64  cagtcttccctttagaatgactgatggtatgctaaggtttttcatagcatatcattatta 123

Query: 119 aaggtgaatacaaat 133
           |||||||||||||||
Sbjct: 124 aaggtgaatacaaat 138
```

Query= SNORA15_NR_002957.1 Length = 133 snoRNA-LBME-db
Sbjct= #236 Length = 206 Blast
CCT6P1

```
Query: 1   gcatggccgaatactgtgtt--tttatcagtagtttacacagccagacaccatgcaaaag 58
           ||||||| ||||||||||||  ||||||||||||||||||||||||||||||||||||||
Sbjct: 4   gcatggctgaatactgtgttcttttatcagtagtttacacagccagacaccatgcaaaag 63

Query: 59  cagtcttccctttagaatgactgatggtatgctaaggtttttcatagcatatcattatta 118
           ||||||||||||||||||||||||||||||||||||||||||||||||||||||||||||
Sbjct: 64  cagtcttccctttagaatgactgatggtatgctaaggtttttcatagcatatcattatta 123

Query: 119 aaggtgaatacaaat 133
           |||||||||||||||
```

```
Sbjct: 124 aaggtgaatacaaat 138
```

Query= SNORA1_NR_003026.1 Length = 130 snoRNA-LBME-db
Sbjct= #244 Length = 164 Blast
LYN

```
Query: 13  gagaatgggcactgttgatcatggtgtccaaaaatagttaatgtggctaaattgagacag 72
           ||||||||||||||||||||| ||||||||||||||||||||||||||||||||||| ||
Sbjct: 147 gagaatgggcactgttgatcaaggtgtccaaaaatagttaatgtggctaaattgagatag 88

Query: 73  gttatgcttccatcacagtatgcatattgcagtggtgacaatgagacctgtaacattt 130
           ||||||||||||||||||||||||||||||||||||||||||||||||||||||||||
Sbjct: 87  gttatgcttccatcacagtatgcatattgcagtggtgacaatgagacctgtaacattt 30
```

Query= SNORD168_NR_145799.1 Length = 58 snoSeeker
Sbjct= #245 Length = 61 Blast
EXT1

```
Query: 1  gcttgatgatgtgttggaggagcaaactggtggcacccaaggctctgttctgaagcag 58
          ||||||||||||||||||||||||||||||||||||||||||||||||||||||||||
Sbjct: 61 gcttgatgatgtgttggaggagcaaactggtggcacccaaggctctgttctgaagcag 4
```

Query= SNORA12_NR_002954.1 Length = 147 snoRNA-LBME-db
Sbjct= #246 Length = 121 Blast
ASAP1

```
Query: 5   gtggtggttttcttttggcacatttgttaagttttcaaatgggcctaactctgccacat 64
           ||||| |||||||||||||||||||||||||||||||||||||||||||| |||||||||
Sbjct: 5   gtggtagttttcttttggcacatttgttaagttttcaaatgggcctaacactgccacat 64

Query: 65  atataatatcggagatggcaaaggcttgtgacg--gagatatctctc 109
            ||| |||  |||| |||||||||| ||||||   ||||||||||||
Sbjct: 65  gtattatactggagttggcaaaggcctgtgacaatgagatatctctc 111
```

Partial sequence; lack of ACA

Query= SNORD149_NR_145785.1 Length = 64 snoSeeker
Sbjct= #247 Length = 142 Blast
ZC3H3

```
Query: 1   gccaagtgatgattttcctcagggcggacccagcagggcctctccttctggattctgaag 60
           ||||||||||||||||||||||||||||||||||||||||||||||||||||||||||||
Sbjct: 142 gccaagtgatgattttcctcagggcggacccagcagggcctctccttctggattctgaag 83

Query: 61  gcca 64
           ||||
Sbjct: 82  gcca 79
```

Query= SNORA51_NR_002981.1 Length = 132 snoRNA-LBME-db
Sbjct= #259 Length = 63 Blast
ZNF41

```
Query: 1  ggcctcctggtgcttaccacaggctgtgttcttacactgactgtatagaaa 51
          |||||||||||||||||||||||||||||||||||||||||||||||||||
Sbjct: 3  ggcctcctggtgcttaccacaggctgtgttcttacactgactgtatagaaa 53
```

Partial sequence; out of range

Query= SCARNA27_NR_003703.1 Length = 126 snoRNA-LBME-db
Sbjct= #261 Length = 132 Blast

IP COIL   KIAA2022

```
Query: 6   tctgcgcaggccccaacctatccatggtttggggctgctatctgcctcataaagcaaagt 65
           |||| ||||| |||||||| | ||||||||||||||||||||||||||||||||||||||
Sbjct: 9   tctgtgcaggacccaacctgtacatggtttggggctgctatctgcctcataaagcaaagt 68

Query: 66  ggagggtttgtcttttggaaaaacaaagtcgttttctcctctgaaaaagcctcctacata 125
           | ||||||||| || || ||| |||||||| ||||||| |||||||||||||||||||||
Sbjct: 69  gaagggtttgttttctgaaaacacaaagtcattttctcttctgaaaaagcctcctacata 128
```

Query= SNORA40_NR_002973.1   Length = 127   snoRNA-LBME-db
Sbjct= #271   Length = 126   Blast

```
Query: 13  tgtttttgtttaac-gtggacaaagacttacagataggtgcaaaaaataaatcctctttt 71
           |||||||||||||| ||| ||||||||||| ||| | |||||||||||||||||||||||
Sbjct: 115 tgtttttgtttaactgtgaacaaagacttatagagaagtgcaaaaaataaatcctctttt 56

Query: 72  gcaacccagaactcattgttcagtatgagttttgatacatataa-aagagatatta 130
           |||||||||||||||||||| |||| | |||||||||||||||| ||| |||||||
Sbjct: 55  gcaacccagaactcattgtttagtaagggttttgatacatataagaagggatatta 1
```

Query= SNORA70B_NR_003707.2   Length = 134   snoSeeker
Sbjct= #275   Length = 181   SnoReport/Blast

IP DYSK

```
Query: 1   tgcagccaattaagctgactgaattcctttccttatggggtccagtgtgcaatggctgt 60
           ||||||||||||||| |||||| |||||||||| |||||| |||||||||||||||||||
Sbjct: 4   tgcagccaattaagccgactgagttcctttcctcatgggggcccagtgtgcaatggctgt 63

Query: 61  aaacagcagcttccttggtagtgtatgcggcctgtttgttgtataggttgctctaaggga 120
           ||| |||||||||||||||||||||||| ||||||||||||||| |||||||||||||||
Sbjct: 64  aaatagcagcttccttggtagtgtatgcagcctgtttgttgtatgggttgctctaaggga 123

Query: 121 ccttggagacag 132
           ||||||||||||
Sbjct: 124 ccttggagacag 135
```

Query= SNORA72_NR_002581.1   Length = 132   snoRNA-LBME-db
Sbjct= #277   Length = 120   Blast

```
Query: 12 attctcgctgttctgattttgtaatagtcaggacaggctaaacattcgctatattaagaccatg 71
               |  |     |||||||||||||||||||||||| |||||| |||||||||||||||||||
Sbjct: 1      tgtcaaaattgattttgtaatagtcaggacaggataaacagtcgctatattaagaccatg 69

Query: 82 catgtgtccccaaacctagttctttccctaggtctggtttcataaatgctggtgataaac 132
           | ||||||| | ||||||||||||||||| ||||| ||| |||||||||||||||||||
Sbjct: 70 tacgtgtccctagacctagttctttccctacgtctgtttttataaatgctggtgataaac 120
```

Query= SNORA40_NR_002973.1   Length = 127   snoRNA-LBME-db
Sbjct= #281   Length = 167   SnoReport/Blast

IP DYSK and Nucleolar localization

```
Query: 1   tgcacttatgtatgtttttgtttaac--gtggacaaagacttacagataggtgcaaaaaa 58
           ||||||| ||||||||||||||||||  ||||||||||||||   |||||||||||||||
Sbjct: 12  tgcacttctgtatgtttttgtttaacttgtggacaaagacttgtggataggtgcaaaaaa 71

Query: 59  taaatcctcttttgcaacccagaactcattgttcagtatgagttttgatacatataagaa 118
           ||| |||||||||||||||  |||||||||||||||||||||||||||||||||||||||
Sbjct: 72  taagtcctcttttgcaacctggaactcattgttcagtatgagttttgatacatataagaa 131
```

```
Query: 119 gggatatta 127
           |||||||||
Sbjct: 132 gggatatta 140
```

Query= SNORD2_NR_002587.1 Length = 70 snoSeeker
Sbjct= #282 Length = 137 Blast

```
Query: 4   tgaaatgatggcaatcatctttcgggactgacctgaaatgaagagaatactcattgctga 63
           |||| ||||| || |||||||| | || |||||  ||||||| |||||||||||||||||
Sbjct: 135 tgaattgatgacagtcatcttttgagagtgacccaaaatgaaaagaatactcattgctga 76

Query: 64  tcacttg 70
           |||||||
Sbjct: 75  tcacttg 69
```

Query= SNORD88C_NR_003069.1 Length = 97 snoRNA-LBME-db
Sbjct= #285 Length = 84 Blast
IP FIB and Nucleolar localization

```
Query  10 ccatgatgtccagcactgggctctgatcacccctgaggacacagtgcaccccaggacctt  69
          |||||||||||||||||||||||||||||| || |||||||||||   ||||| |||| |
Sbjct  5  ccatgatgtccagcactgggctctgatcactccggaggacacagttttccccaagaccat  64

Query  70 tgacacctgggggtctgag  88
           |  |||||||| ||||||
Sbjct  65 ggctacctggggatctgag  83
```

Query= SNORA3B_NR_002977.1 Length = 131 snoRNA-LBME-db
Sbjct= #289 Length = 95 Blast

```
Query: 7  actagagtcacatcctgacacaactcttgtcctggtgtgctagagtactcgaagagaatc 66
          |||||||| ||||||||||||| ||||||||||||||||||||||| |||||   |||||
Sbjct: 87 actagagtaacatcctgacacagctcttgtcctggtgtgctagagttctcga--tgaatc 30

Query: 67 tactggtcttgattcactgg 86
          ||||||||||||||||||||
Sbjct: 29 tactggtcttgattcactgg 10
```

no box defined yet

Query= SNORA38B_NR_003706.2 Length = 132 snoSeeker
Sbjct= #290 Length = 133 Blast

```
Query: 1   cctcctacaaaggcatgtctatagttccttgtctttggacatgtaagaattggaggcaaa 60
           ||||||||||||||||||||||| |||||||||||||||||||||||||||||||| | |
Sbjct: 130 cctcctacaaaggcatgtctataattccttgtctttggacatgtaagaattggagggaca 71

Query: 61  gaaatgtggacttggagaaatctggggccagcttgctctccgcaggctcaagatcaacca 120
           |||||||||||||||||||||||||||||||||| |||  | ||||||||| ||||||||
Sbjct: 70  gaaatgtggacttggagaaatctggggccagctttctcatcacaggctcaacatcaacca 11

Query: 121 tcccacatag 130
           | ||||||||
Sbjct: 10  tgccacatag 1
```

Query= SNORA109_NR_132964.1 Length = 128 snoRNA-LBME-db
Sbjct= #293 Length = 126 Blast
IP DYSK

```
Query: 14  aaaatatactatgaaagtggctttaatacc-ctgaagacacaatagctaagacccaaact 72
```

```
               |||||| |||| |||||||||||||| |   ||||| ||||||||||||||||||||||||
Sbjct: 88   aaaataaactacgaaagtggctttaacatatctgaacacacaatagctaagacccaaact 29

Query: 73   gggattagataccccactatgcttagcc 100
            ||||||||||||||||||||||||||||
Sbjct: 28   gggattagataccccactatgcttagcc 1
```

Partial sequence; lack of ACA

```
---------------------------------
Query= SNORA11D_NR_003711.1      Length = 127     snoRNA-LBME-db
Sbjct= #298                     Length = 94      Blast

Query: 4  tgtgctcagagcagggggcctgaagaatggctcctctgtttacaacacacccaacaggaa 63
           ||||||||||||||||||||||| |||||||||||||||||||||||||||||||||||||
Sbjct: 88 tgtgctcagagcagggggcctgaggaatggctcctctgtttacaacacacccaacaggaa 29

Query: 64 gctggggtcatcgtga 79
             ||||| |||| ||||
Sbjct: 28 tctgggatcattgtga 13
```

Partial sequence; lack of ACA

```
---------------------------------
Query= SNORD11_NR_003031.1      Length = 84      snoRNA-LBME-db
Sbjct= #299                    Length = 88      Blast
IP FIB

Query: 11 atgatttctatttgtttgcctgatttccttttggataatgaaggcatctttagtcactac 70
           ||||||| |||||||||| ||||||||||||||||||||||||| |||||||||||| |
Sbjct: 80 atgattttatttgtttgactgatttccttttggataatgaaggtatctttagtcaccat 21

Query: 71 ctcttctgagaca 83
             |||||||||||
Sbjct: 20 atcttctgagaca 8
```

```
---------------------------------
Query= SNORA3B_NR_002977.1      Length = 131     snoRNA-LBME-db
Sbjct= #300                    Length = 149     Blast
IP DYSK and splicing dependency

Query: 39   tggtgtgctagagtactcgaagagaatctactggtcttgattcactggtgggggcagtcg 98
            ||||||||||  ||||| | |||||||||||||||||||||||||||||||||||||| |
Sbjct: 117  tggtgtgctacggtacttggagagaatctactggtcttgattcactggtgggggcagttg 58

Query: 99   gtgccccgttagtgcccagatcagaaacatac 131
             |||  || ||||||||||||||||||||||
Sbjct: 57   ttgcttcctttagtgcccagatcagaaacatac 25
```

no box defined yet

```
---------------------------------
Query= SNORA70C_NR_003708.4      Length = 136     snoSeeker
Sbjct= #309                     Length = 127     Blast

Query: 10   aattaagccaactgggttcctttcctcatgggggcccattgtgcaatggctgcaaacagc 69
            |||||||||||||| ||| |||||||||  ||| ||   ||||| ||||||||||||||||
Sbjct: 127  aattaagccaactgagtttctttcctcacagggacctggtgtgccatggctgcaaacagc 68

Query: 70   agctttcttggtagtgtatgcagcctgtttgttgtatgggttgctttaagggaccatgga 129
            ||||| ||||  || ||| |||| |||||| ||| |||||||||| ||||||||| ||||
Sbjct: 67   agcttccttgtcagggtacgcagactgtttcttggatgggttgctctaagggaccttgga 8

Query: 130  aacaggc 136
             ||||||
Sbjct: 7    gacaggc 1
```

```
-----------------------------------
Query= SNORA40_NR_002973.1      Length = 127     snoRNA-LBME-db
Sbjct= #310                     Length = 141     SnoReport/Blast

Query: 1   tgcacttatgtatgtttttgtttaac--gtggacaaagacttacagataggtgcaaaaaa 58
           ||||||||||||||||||||||||||  |||||||||||||||  ||||||||||| |||
Sbjct: 138 tgcacttatgtatgtttttgtttaacttgtggacaaagacttatggataggtgcaacaaa 79

Query: 59  taaatcctcttttgcaacccagaactcattgttcagtatgagttttgatacatataagaa 118
           |||||||||||||||||||||||||||||||||||| ||||||||||||||||| | |||
Sbjct: 78  taaatcctcttttgcaacccagaactcattgttcagcatgagttttgatacatacaggaa 19

Query: 119 gggatatt 126
           ||||||||
Sbjct: 18  gggatatt 11

-----------------------------------
Query= SNORA8_NR_002920.1       Length = 139     snoRNA-LBME-db
Sbjct= #314                     Length = 166     SnoReport/Blast

Query: 1   tgcactgcatggtatctgcactcagcagtttacacctgctagggtgttcaaaggtcagtg 60
           |||||||||||| |||||||| ||||||||||| | ||||| ||||||||||||||||||
Sbjct: 3   tgcactgcatggcatctgcacccagcagtttactcttgctaaggtgttcaaaggtcagtg 62

Query: 61  ctatagaaattcagtatctggcatcgttggttttcttggctttgtgcttgttaaacctgg 120
           ||||||||||||||||||||||||| |||||||||||| ||||||||||||||||||| |
Sbjct: 63  ctatagaaattcagtatctggcatcattggttttcttgactttgtgcttgttaaacctag 122

Query: 121 tatttctactgatacagta 139
           |||||||| ||||||||||
Sbjct: 123 tatttctattgatacagta 141

-----------------------------------
Query= SNORA48_NR_002918.1      Length = 135     snoRNA-LBME-db
Sbjct= #315                     Length = 134     Blast

Query: 6   ctgacctgggtagagtggcatctggttggtgatgcccatctcatatcagccagggacaaa 65
           ||||||||||||||||||||||||| || |||    ||||||||||||||||||||||||
Sbjct: 129 ctgacctgggtagagtggcatctggctg-tgacattcatctcatatcagccagggacaaa 71

Query: 66  gcaactccttgttcatcccagcttggcttttgatccgtgcccatgcctggttcatgcctt 125
           ||||| ||||||| ||  ||||||||| |||  || ||||||||||||||||||||||||
Sbjct: 70  gcaaccccttgtttatttcagcttggccttttgtctgtgcccatgcctggttcatgcctt 11

Query: 126 ggacaca 132
           |||||||
Sbjct: 10  ggacaca 4

-----------------------------------
Query= SNORA70B_NR_003707.2     Length = 134     snoRNA-LBME-db
Sbjct= #319                     Length = 107     Blast

Query: 46  gtgtgcaatggctgtaaacagcagcttccttggtagtgtatgcggcctgtttgttgtata 105
           |||||||||||||| | ||||||||||||||||||||||| || ||||||| |||||||
Sbjct: 19  gtgtgcaatggctgcacacagcagcttccttggtagtgtacgcagcctgttggttgtatg 78

Query: 106 ggttgctctaagggaccttggagacaggc 134
           |||||||||||||||||||||||||||||
Sbjct: 79  ggttgctctaagggaccttggagacaggc 107
```

no box defined yet

```
-----------------------------------
Query= SNORA51_NR_002981.1      Length = 132     snoRNA-LBME-db
Sbjct= #320                     Length = 144     Blast
```

```
Query: 1   ggcctcctggtgcttacc-acaggctgtgttcttacactgactgtatagaaagaggaggt 59
           ||||||||||| |||||| |||||||| ||||||||||| ||||| ||||||||||| |
Sbjct: 142 ggcctcctggtacttacccacaggctgcgttcttacactggctgtacagaaagaggagct 83

Query: 60  agagtaaacctaccccatatacacctcagctcaggccctgtgcctggtctgtattgtgaa 119
           ||||||||||||||  |||||||||||||| |||||||||||||||||||||||||||||
Sbjct: 82  agagtaaacctacctaatatacacctcagcccaggccctgtgcctggtctgtattgtgaa 23

Query: 120 tggggg 125
           ||||||
Sbjct: 22  tggggg 17

no box defined yet
-----------------------------------
Query= SNORA40_NR_002973.1      Length = 127     snoRNA-LBME-db
Sbjct= #321                    Length = 141     Blast

Query: 12  atgttttgtttaac--gtggacaaagacttacagataggtgcaaaaaataaatcctctt 69
           ||||||||| |||||  ||||| |||| |||||||| |||||||||||||||||||||||
Sbjct: 127 atgttttgcttaacttgtggataaagtcttacagacaggtgcaaaaaataaatcctctt 68

Query: 70  ttgcaacccagaactcattgttcagtatgagttttgatacatataagaaaggacattt 127
           ||||||||||||||||||||||||||||||||||||||||||||||||| ||| |||
Sbjct: 67  ttgcaacccagaactcattgttcagtatgagttttgatacatataagaagggatatta 10

-----------------------------------
Query= SNORA10_NR_002327.1      Length = 133     snoRNA-LBME-db
Sbjct= #322                    Length = 136     SnoReport/Blast

Query: 1   ggtctctcagctccgcttaaccacacgggtccagtgtgtgcttggcgtgtttcagggag 60
           ||||||||||||| ||||||||||||||||||||||||||||||||||||||||||||||
Sbjct: 3   ggtctctcagctctgcttaaccacacgggtccagtgtgtgcttggcgtgtttcagggag 62

Query: 61  gcagagaaaggctctcctaatgcacgacagacccgcccagaatggcctctctgttcctag 120
           ||||||||||||||||||||||||||||||||||||||||||||||||||||||||||||
Sbjct: 63  gcagagaaaggctctcctaatgcacgacagacccgcccagaatggcctctctgttcctag 122

Query: 121 gagtgcgacaatt 133
           ||||| |||||||
Sbjct: 123 gagtgtgacaatt 135

-----------------------------------
Query= SNORA70C_NR_003708.4     Length = 136     snoSeeker
Sbjct= #330                    Length = 195     SnoReport/Blast

Query: 1   tctgcagccaattaagccaactgggttcctttcctcatggggcccattgtgcaatggct 60
           ||||||||||||||||||||||| |||||||||||  |||||||||| ||||||||||||
Sbjct: 16  tctgcagccaattaagccaactgagttcctttccttgtgggggcccagtgtgcaatggct 75

Query: 61  gcaaacagcagctttcttggtagtgtatgcagcctgtttgttgtatgggttgctttaagg 120
           ||| |||||||||| ||||||||||||  ||||||||| ||||||||||||||| | |||
Sbjct: 76  gcacacagcagcttccttggtagtgtacacagcctgttggttgtatgggttgctctgagg 135

Query: 121 gaccatggaaacaggc 136
           |||| |||| ||||||
Sbjct: 136 gaccttggagacaggc 151

-----------------------------------
Sbjct= #331                    Length = 198     SnoReport
IP FIB and Nucleolar localization

C=11 D=66 17 96 GTGATGT 27 CTGA 82 0.93 C/D box snoRNA
TAAATTAACCCATTTATTACAGGCCAGTGATGTCTCAAAGAGTAGAGGAGCGTCTACTGGTCTTTCAACTCCTTCAGGCT
```

TCTGATGGCGGACTTTACCGTGATAGCGGAGGTGGTATTGTATGTCCAGGCACCGCCAGCCACTGTCTTCACGCGGGAAC
CACAGTGCCAGATCCCCGCAGCTCATCTCTTCATCTTGG

Query= SNORA33_NR_002436.1 Length = 133 snoRNA-LBME-db
Sbjct= #332 Length = 73 Blast

```
Query: 2  agccagccaatgaatctgcttacctgattgtgtttgtgcagacatactttaaaaactggc 61
           |||||| || |||||||||||||||||||||||||||||||| ||| |||||||||||||
Sbjct: 3  agccagtcagtgaatctgcttacctgattgtgtttgtgcagatatattttaaaaactggc 62

Query: 62 aatagta 68
           |||||||
Sbjct: 63 aatagta 69
```

Partial sequence; lack of ACA

Query= SNORA70B_NR_003707.2 Length = 134 snoSeeker
Sbjct= #333 Length = 164 Blast

```
Query: 1   tgcagccaattaagctgactgaattcctttccttatgggggtccagtgtgcaatggctgt 60
            |||| ||||||||||||||||||||||||||||   ||||| |  ||||||||||||||
Sbjct: 161 tgcaaccaattaagctgactgaattcctttcctcccgggggccaggtgtgcaatggctgc 102

Query: 61  aaacagcagcttccttggtagtgtatgcggcctgtttgttgtataggttgctctaaggga 120
            ||||||||||||||||||||||||| || |||||||| ||||||  ||||||||||||||
Sbjct: 101 aaacagcagcttccttggtagtgtacgcagcctgtttcttgtatgcgttgctctaaggga 42

Query: 121 ccttggagacaggc 134
            ||||||||||||||
Sbjct: 41  ccttggagacaggc 28
```

Query= SNORA25_NR_003028.1 Length = 134 snoRNA-LBME-db
Sbjct= #335 Length = 89 Blast

```
Query: 2  ggtcatttcaaagagggcttatgaggctgtgaaacccagagctcttaacgctgtgaccaa 61
           |||||||||||||||| ||| ||||||||||||||| |||||||||||| ||| ||||||
Sbjct: 5  ggtcatttcaaagaggtcttgtgaggctgtgaaaccaagagctcttaacactgcgaccaa 64

Query: 62 agatggaagttctctataggaagcc 86
           ||||||||||||||||||||| |||
Sbjct: 65 agatggaagttctctataggatgcc 89
```

Sbjct= #341 Length = 282 SnoReport

IP DYSK and Nucleolar localization and splicing dependency

C=11 D=66 17 96 GTGATGT 27 CTGA 82 0.93 C/D box snoRNA
CTCAGAGTGGTCAAGTTGAGTTGAAATCTCCTAAGTGTAAGTTGGGTGCTTTATGTTAAGCTACACTCTGGTTCGTCCAA
GTGCACTTTCCACTACACTTACCATGTTACGACTTGTTTCCTCTATATAAATGCGTAGGGGTTTTAGTTAAATATCCTTT
GAAGTATACTTGAGGAGGGTGACGGGCGGTGTGTATGCGCTTCAGGGCCCTGTTCAACTAAGCACTCTACACTTAGTTTA
CTGCTAAATCCACCTTCGACCCTTAAATTTCATAAGCTCCCT

Query= SNORA72_NR_002581.1 Length = 132 snoRNA-LBME-db
Sbjct= #344 Length = 139 SnoReport/Blast

```
Query: 5   gaatattctcgctgttctgattttgtaatagtcaggacaggctaaacattcgctatatta 64
            |||||||||||||||||||||||||||||| ||| ||  |||||||||||||| ||||||
Sbjct: 6   gaatattctcgctgttctgattttgtaataatcacgatgggctaaacattcgcaatatta 65

Query: 65  agaccatgcatgtgtccccaaacctagttctttccctaggtctggtttcataaatgctgg 124
            |||||||| |||| |||| | ||||||||||||||| ||||||||||| |||||||||||
```

```
Sbjct: 66  agaccatgtatgtatccctagacctagttctttccccaggtctggttttataaatgctgg 125

Query: 125 tgataaac 132
            ||||||||
Sbjct: 126 tgataaac 133
```

Query= SNORA75_NR_002921.1 Length = 137 snoRNA-LBME-db
Sbjct= #345 Length = 146 Blast

```
Query: 3   cttctcattgagctcctttctgtctatcagtggcagtttatggattcgcacgagaagaag 62
            |||||||||||||||||||||||| ||   |||||||| ||||||| ||| |||||||||
Sbjct: 143 cttctcattgagctcctttctgtccattgatggcagttgatggatttgcatgagaagaag 84

Query: 63  agagaattcacagaactagcattattttaccttctgtctttacagaggtatatttagctg 122
            |||||||||||||||||||| ||||||||| |||||||||||||||||||||||| ||||
Sbjct: 83  agagaattcacagaactagcgttattttactttctgtctttacagaggtatatttggctg 24

Query: 123 tattgtgagacattc 137
            | |||||||||||||
Sbjct: 23  ttttgtgagacattc 9
```

Query= SNORA109_NR_132964.1 Length = 128 snoRNA-LBME-db
Sbjct= #346 Length = 133 Blast
IP DYSK

```
Query: 4   cagctgaaataaaatatactatgaaagtggctttaa--taccctgaagacacaatagcta 61
            ||||||| |||||||| |||| ||||||||||||||  || | |||| ||||||||||||
Sbjct: 39  cagctgacataaaataaactacgaaagtggctttaacgtatc-tgaacacacaatagcta 97

Query: 62  agacccaaactgggattagataccccactatg 93
            |||||||||||||||||||||||| |||||||
Sbjct: 98  agacccaaactgggattagataccgcactatg 129
```

Partial sequence; lack of ACA

Query= SNORD42B_NR_000013.1 Length = 67 snoRNA-LBME-db
Sbjct= #349 Length = 69 Blast

```
Query: 3  gcatatgatggaaaagttttaatctcctgacacttgtgatgtcttcaaaggaaccactga 62
           ||||||||||||||| | ||||||| |||| |||||||||||||||||||||||||||
Sbjct: 67 gcatatgatggaaaaatcttaatcttctgagacttgtgatgtcttcaaaggaaccacttt 10
```

Query= SNORA31_NR_002967.1 Length = 130 snoRNA-LBME-db
Sbjct= #351 Length = 57 Blast

```
Query: 81  ccttcgctttccgtctgtctaattgtggca-gctgagattgaatagaggaatacagga 133
                | ||||| |||||||| ||||| |||  ||||||||||||||||||||||||||
Sbjct: 1   ----cactttctgtctgtctgattgtagcagcctgagattgaatagaggaatacagga  52
```

Query= SNORA18_NR_002959.1 Length = 132 snoSeeker
Sbjct= #356 Length = 95 Blast

```
Query: 46  ttggaaacgcctcatagagtaactctgtggttttactttactcacaggactattgttaga 105
            ||||||| ||||||||||||||||||||||||||| ||||| ||||||||||| |||| |
Sbjct: 7   ttggaaatgcctcatagagtaactctgtggttttattttacccacaggactatggttaaa 66

Query: 106 tctgtgggaaggaattacaagacagtt 132
            | |||||||| ||| ||||||||||||
Sbjct: 67  tatgtgggaaagaactacaagacagtt 93
```

```
-----------------------------------
Sbjct= #357                     Length = 68      SnoReport
IP FIB

C=7 D=58 1 69 ATGATGG 7 CTGA 58 0.57 C/D box snoRNA
GTCCTGATGATGGTAGTCAAGACTGCACACTGTTGGTGTGCGGAGCATTTCCCCACTCTGATTGGGCT

-----------------------------------
Query= SNORA27_NR_002575.1      Length = 126     snoRNA-LBME-db
Sbjct= #362                     Length = 133     SnoReport/Blast

Query: 4   cccctttttcactttgccagttggacttatgtctttattggtcattcaagtggggcaaagg 63
           ||||| ||||||||||||  |||| || ||||||||||| ||||| || |||||||||||
Sbjct: 6   cccctgttcactttgccaaatggatttgtgtctttattgctcatt-aattggggcaaagg 64

Query: 64  aaatatcctttttaaaactcaggcaaactgggtgtttgtc-tgtatcctgtcagaggaaac 122
           |||||||||||||||||||||||||||||| ||||||||| ||||||||||||||||||||
Sbjct: 65  aaatatcctttttaaaactcaggcaaactgagtgtttgtcttgtatcctgtcagaggaaag 124

Query: 123 aaat 126
           ||||
Sbjct: 125 aaat 128

-----------------------------------
Query= SNORA70C_NR_003708.4     Length = 136     snoSeeker
Sbjct= #363                     Length = 164     SnoReport/Blast

Query: 1   tctgcagccaattaagccaactgggttcctttcctcatggggcccattgtgcaatggct 60
           |||||||||||||||||| |||| |||||||||||||||||||||| |||||||| |||
Sbjct: 2   tctgcagccaattaagccgactgagttcctttcctcatggggcccagtgtgcaatcgct 61

Query: 61  gcaaacagcagctttcttggtagtgtatgcagcctgtttgttgtatgggttgctttaagg 120
           |||||||||||||| ||||||||| |||||||||||||| ||||| |||||||| || ||
Sbjct: 62  gcaaacagcagcttccttggtagtatatgcagcctgtttattgtacgggttgctctacgg 121

Query: 121 gaccatggaaacaggc 136
           |||| |||| ||||||
Sbjct: 122 gaccttggagacaggc 137

-----------------------------------
Query= SNORD28B_NR_145751.1     Length = 75      snoSeeker
Sbjct= #367                     Length = 72      Blast

Query: 2  tcagatgatttgaattgataagctgaggttctgtgaggaatgacagttaagagcatgtta 61
          ||||||||||||||||||||||||||||||||||||||||||||||||||||||||||||
Sbjct: 1  tcagatgatttgaattgataagctgaggttctgtgaggaatgacagttaagagcatgtta 60

Query: 62 aagttgttatga 73
          ||||||||||||
Sbjct: 61 aagttgttatga 72

-----------------------------------
Query= SNORA63_NR_002586.1      Length = 135     snoRNA-LBME-db
Sbjct= #370                     Length = 90      Blast

Query: 59  taaaataaagttgtttcttaactatacctgtctgctattctcctgtagcagccagggacg 118
           |||||||||||||||||||||||||||||||||||||||||||||||||| ||||||| |
Sbjct: 7   taaaataaagttgtttcttaactatacctgtctgctattctcctgtagcaaccagggatg 66

Query: 119 cttggtctcatacatgt 135
            ||||| ||||||||||
Sbjct: 67  attggtttcatacatgt 83

-----------------------------------
```

Query= SNORA26_NR_003016.1 Length = 122 snoRNA-LBME-db
Sbjct= #373 Length = 63 Blast

```
Query: 65   taaaacccagagaagagactggaaagctcctctttggatcctgtctggagtcaca 119
            ||||||||| |||||||| || ||| || ||||||||||||||||||||||||||
Sbjct: 5    taaaacccaaagaagagattgtaaaacttctctttggatcctgtctggagtcaca 59
```

Query= SNORA70B_NR_003707.2 Length = 134 snoSeeker
Sbjct= #374 Length = 135 SnoReport/Blast

```
Query: 1    tgcagccaattaagctgactgaattcctttccttatggggtccagtgtgcaatggctgt 60
            ||||||||||| ||||||  || ||||||||||  |||||  |||||||||||  ||||
Sbjct: 3    tgcagccaattgagctgatggagttcctttcctcctggggc-ccagtgtgcaaaagctgc 61

Query: 61   aaacagcagcttccttggtagtgtatgcggcctgtttgttgtataggttgctctaaggga 120
            ||||||||||||||||||||||||| || |||||||| || ||| |||||||||||||||
Sbjct: 62   aaacagcagcttccttggtagtgtacgcagcctgtttcttatatgggttgctctaaggga 121

Query: 121 ccttggagacag 132
            ||||||||||||
Sbjct: 122 ccttggagacag 133
```

Query= SNORA67_NR_002912.1 Length = 137 snoRNA-LBME-db
Sbjct= #375 Length = 94 Blast

```
Query: 1  atccaaggtgattccctctccaaggggacat-cagtgcc--tctcaggaaagtagcagct 57
           |||||||| ||||||||||||||||| |||| |||||||  ||||||||||||| || |
Sbjct: 94 atccaaggggattccctctccaagggaacatgcagtgcccctctcaggaaagtaacaacc 35

Query: 58 tggaatagaatctggcatgcctaaggcctttg 89
           |||||||||||||||||||||||||| |||||
Sbjct: 34 tggaatagaatctggcatgcctaaggtctttg 3
```

Query= RNU6ATAC_NR_023344.1 Length = 105
Sbjct= #378 Length = 125 Blast

```
Query   4     AAGGATGGTCAGATGCCACGAAGTAGGTGGCAATGCCTTAACTGTATGTGTGTTGTCAGG   63
              || |||||| |||||||||||||||||||||||||||||||| ||||| |||||||||||
Sbjct   122   AACGATGGTTAGATGCCACGAAGTAGGTGGCAATGCCTTAACCGTATGCGTGTTGTCAGG   63

Query   64    CTTGAGGGCCTCTTTCATCCTTGTCAAGAGTTGTGCCAA   102
              |  ||||||||||| ||||||||||||| |  |||| ||
Sbjct   62    CCCGAGGGCCTCTTCCATCCTTGTCAAGGGGAGTGCTAA   24
```

Query= SNORA72_NR_002581.1 Length = 132 snoRNA-LBME-db
Sbjct= #379 Length = 64 Blast

```
Query: 1  ctgcgaatattctcgctgttctgattttgtaatagtcaggacaggctaaacattcgctat 60
           |||| |||||||||  || ||||||||||||||||||||||||| ||||||||| |||||
Sbjct: 63 ctgccaatattctcattgctctgattttgtaatagtcaggacagcctaaacatttgctat 4

Query: 61 att 63
           |||
Sbjct: 3  att 1
```

Query= SNORA40_NR_002973.1 Length = 127 snoRNA-LBME-db
Sbjct= #380 Length = 141 Blast

```
Query: 1   tgcacttatgtatgtttttgtttaac--gtggacaaagacttacagataggtgcaaaaaa 58
```

```
           |||||||||||||||||||||||||   |||||||||||||||  |||| || |||||||
Sbjct: 138 tgcacttatgtatgtttttgtttaaactgtggacaaagacttatggataagtacaaaaaa 79

Query: 59  taaatcctcttttgcaacccagaactcattgttcagtatgagttttgatacatatagccag 119
           |||||||||||||||||||||||||||||||||||||||||||||| ||||||||||   ||
Sbjct: 78  taaatcctcttttgcaacccagaactcattgttcagtatgagttttaatacatataagaag 19

Query: 120 ggaaatta 128
           ||||||||
Sbjct: 18  ggatatta 10
```

Query= SNORA31B_NR_145985.1 Length = 134 snoRNA-LBME-db

Sbjct= #381 Length = 80 Blast

```
Query: 5  atctatttgacagacctggagcagttgctatctgctgctatggtttccaccacagatgca 64
          |||||||||||||||||||||||||| || |||||||||| |||||||| ||||||||||
Sbjct: 75 atctatttgacagacctggagcagttactgtctgctgctaaggtttccactacagatgca 16

Query: 65 ag 66
          ||
Sbjct: 15 ag 14
```

Partial sequence; out of range

Query= SNORA67_NR_002912.1 Length = 137 snoRNA-LBME-db

Sbjct= #385 Length = 63 Blast

```
Query: 79  taaggcctttggggaactgggatgcttatttcctctgccttccttggctgcc 130
           ||||| ||||||||||| |||||||||||||||||||||||||||| |||||
Sbjct: 59  taaggtctttggggaacagggatgcttatttcctctgccttccttgtctgcc 8
```

Partial sequence; out of range

Query= SNORA48_NR_002918.1 Length = 135 snoRNA-LBME-db

Sbjct= #386 Length = 138 Blast

```
Query: 3   tccctgacctgggtagagtggcatctggttggtgatgcccatctcatatcagccagggac 62
           |||||||||||||||||||||||||  ||||||| |||||||||||||||||||||||||
Sbjct: 135 tccctgacctgggtagagtggcatccagttggtggtgcccatctcatatcagccagggac 76

Query: 63  aaagcaactccttgttcatcccagcttggcttttgatccgtgcccatgcctggttcatgc 122
           |||||||| |||||||| |||||||||||||||| ||| ||||| |||||||||||||||
Sbjct: 75  aaagcaaccccttgttcctcccagcttggcttttcatctgtgcctatgcctggttcatgc 16

Query: 123 cttggacacat 133
           |||||||||||
Sbjct: 15  cttggacacat 5
```

Query= SNORD45B_NR_002748.1 Length = 71 snoRNA-LBME-db

Sbjct= #391 Length = 77 Blast

```
Query: 1  ggtcaatgatgtaatggcatgtattagctgaatctaaagttgatgtgagttctagaatta 60
          |||||||||||||||||||||||||||||||||| |||||||| |||| ||||| |||||
Sbjct: 2  ggtcaatgatgtaatggcatgtattagctgaatccaaagttgaagtgaattctaaaatta 61

Query: 61 cac 63
          |||
Sbjct: 62 cac 64
```

Query= SNORA40_NR_002973.1 Length = 127 snoRNA-LBME-db

Sbjct= #392 Length = 105 Blast

```
Query: 47  aggtgcaaaaaataaatcctcttttgcaacccagaactcattgttcagtatgagttttga 106
           |||||||||||||||||  |||||||| || |||||||||||||||||||||||||||||
Sbjct: 25  aggtgcaaaaaataaataatcttttgccactcagaactcattgttcagtatgagttttga 84

Query: 107 tacatataagaagggatatta 127
           |||| ||||||||| ||||||
Sbjct: 85  tacacataagaaggaatatta 105
```

Query= SNORA8_NR_002920.1 Length = 139 snoRNA-LBME-db
Sbjct= #396 Length = 169 SnoReport/Blast

```
Query: 1   tgcactgcatggtatctgcactcagcagtttacacctgctagggtgttcaaaggtcagtg 60
           |||||||| |||||||||||||||||||||||| ||||||||||||||||||||||||||
Sbjct: 3   tgcactgcgtggtatctgcactcagcagtttactcctgctagggtgttcaaaggtcagtg 62

Query: 61  ctatagaaattcagtatctggcatcgttggtttcttggctttgtgcttgttaaacctgg 120
           | |||||||| ||||||||||  || ||||||||||||||||||||||||||||||||||
Sbjct: 63  ccatagaaatccagtatctggtttcattggtttcttggctttgtgcttgttaaacctgg 122

Query: 121 tatttctactgatacag 137
           |||||||| ||||||||
Sbjct: 123 tatttctattgatacag 139
```

Query= SNORA18_NR_002959.1 Length = 132 snoSeeker
Sbjct= #397 Length = 109 Blast
IP DYSK and Nucleolar localization and splicing dependency

```
Query: 26  ttcctgtagcctgcacatcgttggaaacgcctcatagagtaactctgtggttttacttta 85
           |||||||||||||||| || || ||||| ||||||| |||||||  ||||||||||||||
Sbjct: 4   ttcctgtagcctgcacgtccttagaaacacctcataaagtaact--gtggttttacttta 61

Query: 86  ctcacaggactattgttagatctgtgggaaggaattacaagacagtt 132
           ||||||||||||||| ||||||| |||||| ||| || |||||||||
Sbjct: 62  ctcacaggactattgctagatctatgggaaagaactataagacagtt 108
```


1. Lestrade L, Weber MJ (2006). snoRNA-LBME-db, a comprehensive database of human H/ACA and C/D box snoRNAs. Nucleic Acids Res. 34, D158-D162.

2. Altschul SF, Madden TL, Schaffer AA, Zhang J, Zhang Z, Miller W, Lipman DJ (1997). Gapped BLAST and PSI-BLAST: a new generation of protein database search programs. Nucleic Acids Res. 25, 3389-3402.

3. Altschul SF, Gish W, Miller W, Myers EW, Lipman DJ (1990). Basic local alignment search tool. J Mol Biol. 215, 403-410.

4. Yang JH, Zhang XC, Huang ZP, Zhou H, Huang MB, Zhang S, Chen YQ, Qu LH (2006). snoSeeker: an advanced computational package for screening of guide and orphan snoRNA genes in the human genome. Nucleic Acids Res. 34, 5112-5123.

5. Jorjani H, Kehr S, Jedlinski DJ, Gumienny R, Hertel J, Stadler PF, Zavolan M, Gruber AR (2016). An updated human snoRNAome. Nucleic Acids Res. 44, 5068-5082.

6. de Araujo Oliveira JV, Costa F, Backofen R, Stadler PF, hado Telles Walter ME, Hertel J (2016). SnoReport 2.0: new features and a refined Support Vector Machine to improve snoRNA identification. BMC Bioinformatics. 17, 464-
